# Qifu Huazhuo formula for gout recurrence prevention: an interim analysis combining clinical outcomes with proteomic and metabolomic profiling

**DOI:** 10.3389/fphar.2025.1642121

**Published:** 2025-10-21

**Authors:** Zhengdong Shen, Fangjie Zhu, Yongliang Chu, Haiyu Wang, Maojie Wang, Xiumin Chen, Yunting Xiao, Yongqiang Yang, Runyue Huang

**Affiliations:** ^1^ State Key Laboratory of Traditional Chinese Medicine Syndrome, The Second Affiliated Hospital of Guangzhou University of Chinese Medicine (Guangdong Provincial Hospital of Chinese Medicine), The Second Clinical Medical College of Guangzhou University of Chinese Medicine, Guangzhou, China; ^2^ Department of Rheumatology, Zhuhai Branch of The Second Affiliated Hospital of Guangzhou University of Chinese Medicine, Zhuhai, China; ^3^ Guangzhou University of Chinese Medicine, Guangzhou, China; ^4^ Department of Rheumatology and Hematology, The First Clinical Medical College of Shaanxi University of Traditional Chinese Medicine, Xianyang, China; ^5^ Guangdong Provincial Key Laboratory of Clinical Research on Traditional Chinese Medicine Syndrome, Guangzhou, China; ^6^ State Key Laboratory of Dampness Syndrome of Chinese Medicine, The Second Affiliated Hospital of Guangzhou University of Chinese Medicine (Guangdong Provincial Hospital of Chinese Medicine), Guangzhou, China

**Keywords:** TCM, gout recurrence, multi-omics, rct, lysosomes

## Abstract

**Purpose:**

Gout is a chronic disease caused by the deposition of monosodium urate crystals in joints and tissues. The Qifu Huazhuo (QFHZ) Formula has shown effectiveness and safety in the management of gout. However, the role of QFHZ in the mitigation of gout needs to be further explored.

**Materials and methods:**

UHPLC-MS/MS was used to identify potential metabolites of QFHZ. Then we conducted a midpoint evaluation of the clinical study on the treatment of gout with QFHZ formula. The clinical study was a monocenter, open-label, randomized controlled trial. Eligible participants were allocated to TM, WM and TWM three groups in random. Participants in TM, WM and TWM group were received QFHZ (250 mL/dose, twice daily, oral), febuxostat (40 mg/dose, once daily, oral) and combination of febuxostat (40 mg/dose, once daily, oral) with QFHZ (250 mL/dose, twice daily, oral) for 12 weeks respectively. The primary efficacy endpoint is the percentage change in serum uric acid. The secondary efficacy endpoint include frequency of gout attacks, the change in estimated glomerular filtration rate (eGRF) and serum creatinine from baseline. Proteomic and metabolomic profiling was performed on paired pre- and post-treatment plasma samples.

**Results:**

Pharmacological studies have indicated that QFHZ contains 14 major metabolites. Clinical research has found that, TM can reduce the frequency of gout attacks compared to WM (*p* = 0.0006), while no significant differences were observed in the percentage change of serum uric acid levels across the three groups. Combined with proteomics and metabolomics analysis, it was discovered that QFHZ may regulate neutrophil extracellular trap (NET) formation, complement, lysosomes, phagosomes, and ferroptosis related biomolecules.

**Conclusion:**

QFHZ shows distinct advantages in preventing gout recurrence over urate-lowering therapy alone, with multi-omics profiling revealing its potential multi-target effects. Future studies should validate these findings in larger cohorts and further elucidate the underlying molecular mechanisms.

**Clinical Trial Registration:**

https://www.chictr.org.cn/showproj.html?proj=198890, identifier ChiCTR2300073188.

## Introduction

Gout is a chronic disease caused by the deposition of monosodium urate crystals in joints and tissues. In China, the prevalence of gout is approximately 1%–3%, with an increasing prevalence, particularly among younger populations (Xu et al., 2023). Studies have shown that Traditional Chinese Medicine (TCM) is both safe and effective in lowing uric acid, treating acute gout and gouty nephropathy (Liu et al., 2023a; Liu et al., 2024a). Mechanistically, botanical drugs or their extracts possess various pharmacological effects, including anti-inflammatory (Yan et al., 2024), anti-reactive oxygen species (ROS) (Huang et al., 2024), inhibition of uric acid production and promotion of its excretion (Wang et al., 2025), and endoplasmic reticulum stress-reducing (Pi et al., 2024). Therefore, TCM has gained increasing recognition and utilization as a supplementary or alternative treatment for gout (Liu et al., 2024b; Zeng et al., 2024). However, there is still a lack of clinical research on TCM treatment for gout, especially in preventing gout recurrence. Our previous study has demonstrated that Qifu Huazhuo (QFHZ) Formula reduces serum uric acid levels and decreases the frequency of gout recurrence (Xia et al., 2020). These findings suggest that QFHZ is likely to exert multifaceted therapeutic effects for gout. Multi-omics approaches integrate data from different levels of biological processes to provide a comprehensive insight into organismal function (Fu et al., 2023). Therefore, this study aims to characterize the chemical profile of QFHZ and preliminarily explore its potential roles in gout through a randomized controlled trial integrated with multi-omics analyses.

## Materials and methods

### Preparation of drugs

The botanical drugs, dosage, and production batch number of QFHZ are presented in Table 1. QFHZ comprises *Astragalus mongholicus* Bunge [Fabaceae; Astragali radix], *Smilax glabra* Roxb. [Smilacaceae; Smilacis glabrae rhizoma], *Plantago asiatica* L. [Plantaginaceae; Plantaginis asiaticae herba], *Epimedium brevicornu* Maxim. [Berberidaceae; Epimedii brevicornis folium], *Atractylodes macrocephala* Koidz. [Asteraceae; Atractylodis macrocephalae rhizoma], *Cornus officinalis* Sieb. et Zucc. [Cornaceae; Cornus officinalis fructus], *Salvia miltiorrhiza* Bunge [Lamiaceae; Salviae miltiorrhizae radix et rhizoma], *Eucommia ulmoides* Oliv. [Eucommiaceae; Eucommiae ulmoidis cortex], *Clematis chinensis* Osbeck [Ranunculaceae; Clematidis chinensis radix et rhizoma], *Morinda officinalis* How [Rubiaceae; Morindae officinalis radix] and *Dioscorea hypoglauca* Palibin [Dioscoreaceae; Dioscoreae hypoglaucae rhizoma]. The botanical drugs were sourced from Guangdong Provincial Hospital of Chinese Medicine in Guangdong, China (The test reports for various batches of the botanical drug can be found in the Supplementary Figure S1). The drug-solvent ratio (DSR) is 225 g of herbal material to 2,600 mL of solvent, which can be expressed as a ratio of 1 : 11.6. The cooking process is carried out in four steps. First, the botanical drugs are placed in a casserole and covered with 600 mL of cold water. They are then soaked for 30 min, with the exact botanical drug-to-water ratio specified to ensure standardization. Next, 1,200 mL of cold water is added to the soaked botanical drugs, and the mixture is brought to a boil and simmered for 40 min. The decoction is filtered twice through a fine mesh sieve or cloth to remove any residual plant material. For the second boiling, 800 mL of hot water is added to the boiled botanical drugs and the mixture is brought to a boil again and simmered for 30 min. The decoction is filtered twice to collect the liquid, ensuring all plant particles are removed. Finally, the decoctions from the first and second boilings are combined. It is recommended to mix the combined decoction twice within a 24-h period, with each mixing involving 250 mL of the decoction, to achieve a homogeneous solution. Febuxostat, with the trade name Youlitong, is produced by Jiangsu Wanbang Biochemical Pharmaceutical Group Co., Ltd., and has the national drug approval number H20130058.

**TABLE 1:** The components of QFHZ.

Latinname	Chinesename	Medicinalpart	Lotno	Dailyadultdose(g)
*Astragalus mongholicus*	Huangqi	Dry roots	230800971	30
*Smilax glabra*	Tufuling	Dried rhizomes	230500711	30
*Plantago asiatica*	Cheqiancao	whole herb	23040204	30
*Epimedium brevicornu*	Yinghuanghuo	Dry leaves	2304001	10
*Atractylodes macrocephala*	Baizhu	Dried rhizomes	230201	20
*Cornus officinalis*	Shanzhuyu	Dry and mature fruit pulp	230504121	10
*Salvia miltiorrhiza*	Danshen	Dry roots and rhizomes	230603771	30
*Eucommia ulmoides*	Duzhong	Dry bark	2303001	15
*Clematis chinensis*	Weilingxian	Dry roots and rhizomes	2301001	15
*Morinda officinalis*	Bajitian	Dry roots	230705481	15
*Dioscorea hypoglauca*	Fenbixie	Dried rhizomes	230502241	20

### Pharmacochemical analysis of QFHZ in serum

#### Sample preparation

Adult male SD rats (weight 300 + 20 g) were purchased from the Weitonglihua Experimental Animal Technology Co., Ltd of Guangdong (Guangzhou, China). All rats were kept under a standard laboratory conditions (23 °C–25 °C, 12 h light/dark cycles, 55% ± 5% humidity) and fed approved ordinary chow for at least 7 days along with free access to water. Animal studies were conducted in compliance with the ARRIVE guidelines. The experimental protocols all followed the Guidelines for the Care and Use of Laboratory Animals and were also approved by the Shenzhen Lingfu Tuopu Biotechnology Co., Ltd. Experimental Animal Management and Use Committee (Approval No. TOP-IACUC-2024–0121). Rats were randomly divide into a blank group and a treatment group with 3 rats in each group. The treatment group is given QFHZ (60.75 g/kg) by gavage based on the weight of the rats, while the blank group is given the same dose of physiological saline by gavage. After 3 days of continuous administration, fasting without water for 12 h was performed until 5 days. Blood was collected from the abdominal aorta after feeding QFHZ and placed in an EDTA anticoagulant tube. After being left to stand for 30 min, blood samples were centrifuged to separate serum. The same group’s serum was then combined in equal parts and kept at −80 °C for further examination. The serum samples and QFHZ aqueous extract were thawed on ice. After 30 s vortex, the samples were centrifuged at 12,000 rpm for 15 min at 4 °C. 300 μL of supernatant was transferred to a fresh tube and 1,000 μL of extracted solution containing 10 μg/mL of internal standard was added, then the samples were sonicated for 5 min in ice-water bath. After placing 1 h in −40 °C, the samples were centrifuged at 12,000 rpm for 15 min at 4 °C. The supernatant was carefully filtered through a 0.22 μm microporous membrane, then take 30 μL from each sample and pooling as QC samples. Store at −80 °C until the UHPLC- MS/MS analysis.

#### UHPLC- MS/MS analysis conditions

UHPLC- MS/MS analysis was performed on an UHPLC system (Vanquish, Thermo Fisher Scientific) with a Waters UPLC BEH C18 column (1.7 μm 2.1*100 mm). The sample injection volume was set at 5 μL. The flow rate was set at 0.5 mL/min. The mobile phase consisted of 0.1% formic acid in water (A) and 0.1% formic acid in acetonitrile (B). The multi-step linear elution gradient program was as follows: 0–11 min, 85%–25% A; 11–12 min, 25%–2% A; 12–14 min, 2%–2% A; 14–14.1 min, 2%–85% A; 14.1–15 min, 85%–85% A; 15–16 min, 85%–85% A. An Q Exactive Focus mass spectrometer coupled with an Xcalibur software was employed to obtain the MS and MS/MS data based on the IDA acquisition mode. During each acquisition cycle, the mass range was from 100 to 1,500,and the top three of every cycle were screened and the corresponding MS/MS data were further acquired. Sheath gas flow rate: 45 Arb, Aux gas flow rate: 15 Arb, Capillary temperature: 400 °C. Full ms resolution: 70,000, MS/MS resolution: 17,500, Collision energy: 15/30/45 in NCE mode, Spray Voltage: 4.0 kV (positive) or −3.6 kV (negative). The raw mass spectrometry data were imported using XCMS software, followed by retention time correction, peak detection, peak extraction, peak integration, and peak alignment. Compounds were identified by matching against an in-house MS/MS spectral database and applying fragmentation pattern-based matching for peaks with MS/MS data.

### Study design of clinical trial

Participates were screened from a 12-week, monocenter, open-label, randomized controlled trial, which was conducted in the Second Affiliated Hospital of Guangzhou University of Chinese Medicine (Guangdong Provincial Hospital of Chinese Medicine). All the participants were provided written informed consent, and the protocol was first approved by the Medical Ethics Committee of the Second Affiliated Hospital of Guangzhou University of Chinese Medicine (ZF2023-121-01) and subsequently registered with the World Health Organization clinical trial registry (no. ChiCTR2300073188).

### Participants

Participants for this study were recruited from the outpatient rheumatology department at the Second Affiliated Hospital of Guangzhou University of Chinese Medicine (Guangdong Provincial Hospital of Chinese Medicine) in Guangzhou, China. Participants were screened during their clinic visits according to the predefined inclusion and exclusion criteria, detailed later in the manuscript. Eligible participants were invited to join the trial, and those expressing interest were further approached by the trial coordinator, who provided a thorough explanation of the study’s purpose and requirements. Written informed consent was obtained from all participants prior to enrollment.

### Inclusion/exclusion criteria

Participants should meet the following criteria in this study: 1) diagnosed with gout based on the classification diagnostic criteria of 2015 American College of Rheumatology (ACR) (Neogi et al., 2015) and yang deficiency in Spleen and kidney based on the diagnostic criteria of Traditional Chinese Medicine Diagnosis and Treatment Plans for 95 Diseases in 22 Majors (State Administration of Traditional Chinese Medicine, 2010). 2) During the screening period, the uric acid level is higher than 420 μmol/L 3) Signed an informed consent form and be able to adhere to the research visit schedule. Participants were excluded from this trial if they: 1) with cardiovascular diseases such as coronary heart disease, heart failure, and arrhythmia; 2) had a history of allergies to relevant experimental drugs (including experimental drugs, control drugs, etc.); 3) the ALT value was greater than 1.5 times the upper limit of the normal value; 4) had a serum creatinine level of <30 mL/min/1.73 m^2^; 5) used antibiotics nearly 3 months ago; 6) had been using colchicine within the past 2 weeks; 7) pregnant women, lactating women, and men/women who plan to conceive in the near future; 8) with intestinal diseases or diarrhea 3 weeks ago; 9) with other autoimmune diseases combined; 10) with poor blood pressure control (BP > 140/90 mmHg), poor blood glucose control (HbA1c > 7%), and poor blood lipid control (total cholesterol >5.2 mmol/L, triglycerides >1.7 mmol/L); 11) was using thioprine or azathioprine; 12) the reasons might cause interference with the experimental results based on physician’s evaluation and judgment.

### Interventions

Eligible participants were randomly assigned to three groups (TM, WM, and TWM) in a 1:1:1 allocation ratio. Participants in TM, WM and TWM group were received QFHZ (250 mL/dose, twice daily, oral), febuxostat (40 mg/dose, once daily, oral) and combination of febuxostat (40 mg/dose, once daily, oral) with QFHZ (250 mL/dose, twice daily, oral) for 12 weeks respectively. All participates were suggested to reduce quantity of meat and alcohol. Plasma were collected before and after treatment from participants.

### Study endpoints

The primary efficacy endpoint is the percentage change in serum uric acid from baseline at the final visit. The secondary efficacy endpoints included 1) the frequency of gout attacks within the 12 weeks of before and after treatment (defined as times of needed to apply colchicine or NSAIDs), 2) the change in estimated glomerular filtration rate (eGRF) from baseline, 3) changes in ultrasound imaging from baseline, 4) patient reported outcomes, and 5) traditional Chinese medicine wet disease information collection and scoring. The interim analysis primarily focused on the effects of QFHZ on serum uric acid levels, gout recurrence, and renal function. Therefore, in this study, statistical analysis was performed only for the first three clinical outcome measures.

### Method of midpoint clinical trial and employment of sample

This study is a three-arm randomized controlled trial. Based on the methodology described by Browne (1995), a sample size of 30 participants per group was determined for this study (Browne, 1995) (Browne, R.H. On the use of a pilot study for sample size determination. Statistics in Medicine, 1995, 14: 1933–1940). To account for a potential dropout rate of 20%, 36 participants were recruited for each group. An interim analysis was conducted when half of the participants (n = 54) have completed follow-up. The primary objectives of the interim analysis were to evaluate safety, efficacy, and sample size adequacy to ensure trial progression. Using the Pocock method with one interim analysis, the alpha value was adjusted to 0.029 for each stage (interim and final) while controlling the overall Type I error at 0.05. A Bonferroni correction further adjusted the alpha to 0.0097 (0.029/3) for pairwise comparisons. Additionally, we randomly selected 10 patients per group who completed the 12-week follow-up and analyzed their pre- and post-treatment plasma samples for omics profiling. Given the exploratory nature of within-group omics comparisons, no additional alpha adjustment was applied.

### Randomisation and blinding

The randomization sequence was generated using the PROC PLAN procedure in SAS 9.2 by the methodology team. The allocation results were disseminated through the Interactive Web Response System (IWRS) for Clinical Research at The Second Affiliated Hospital of Guangzhou University of Chinese Medicine. Two trained researchers were involved in enrollment and assignment: one was responsible for screening and enrolling eligible participants, while the other was tasked with disclosing the assigned interventions. Participants were assessed the outcomes and clinical parameters at baseline and week 12 by trained researchers who did not know the treatments in the trail.

### Sample collection

Blood samples from participants were collected in EDTA anticoagulant tubes before and after treatment. The tubes were centrifuged at 10,000 rpm for 10 min to obtain plasma, which was then stored at −80 °C until omics analysis.

### Proteomics analysis

The dried peptide samples were reconstituted with mobile phase A (100% H2O, 0.1% FA), centrifuged at 20,000 g for 10 min, and the supernatant was taken for injection. Separation was performed by Bruker nanoElute. The sample was first enriched in the trap column and desalted, and then entered a tandem self-packed C18 column (75 μm internal diameter, 1.8 μm column size, 25 cm column length), and separated at a flow rate of 300 nL/min by the following effective gradient: 0 min, 2% mobile phase B (100% ACN, 0.1% FA); 0∼45 min, mobile phase B linearly increased from 2% to 22%; 45∼50 min, mobile phase B rose from 25% to 35%; 50∼55 min, mobile phase B rose from 35% to 80%; 55∼60 min, 80% mobile phase B. The nanoliter LC separation end was directly connected to a tandem mass spectrometer timsTOF Pro for DDA (Data dependent Acquisition) mode detection. The main parameters were set: ion source voltage was set to 1.6 kV, ion mobility range was 0.6–1.60 0.6–1.60 V·S/cm^2^; MS1 mass spectrometer scanning range was 302∼1,077 m/z and the peak intensity above 2,500 can be detected; The 302∼1,077 m/z was divided into 4 steps, and each step was divided into 8 Windows. A total of 32 Windows was used for continuous window fragmentation and information collection. The fragmentation mode was CID, the fragmentation energy was 10 eV, and the mass width of each window was 25. The cycle time of a DIA scan was 3.3 s. Then, mProphet algorithm was used to complete analytical quality control, thus obtaining a large number of reliable quantitative results. This pipeline also performed GO, COG, Pathway functional annotation analysis and time series analysis. Based on the quantitative results, the differential proteins between comparison groups were found, and finally function enrichment analysis, protein-protein interaction (PPI) and subcellular localization analysis of the differential proteins were performed by Dr. TOM platform developed by BGI.

### Metabolomics analysis

This experiment used Waters UPLC I-Class Plus (Waters, United States) tandom Q Exactive high resolution mass spectrometer (Thermo Fisher Scientific, United States) for separation and detection of metabolites. Chromatographic conditions: Chromatographic separation was performed on a Waters ACQUITY UPLC BEH C18 column (1.7 μm, 2.1 mm × 100 mm, Waters, United States), and the column temperature was maintained at 45 °C. The mobile phase consisted of 0.1% formic acid (A) and acetonitrile (B) in the positive mode, and in the negative mode, the mobile phase consisted of 10 mM ammonium formate (A) and acetonitrile (B). The gradient conditions were as follows: 0–1 min, 2% B; 1–9 min, 2%–98% B; 9–12 min, 98% B; 12–12.1 min, 98% B to 2% B; and 12.1–15 min, 2% B. The flow rate was 0.35 mL/min and the injection volume was 5 μL. Mass spectrometry conditions: Using Q Exactive (Thermo Fisher Scientific, United States) perform primary and secondary mass spectrometry data acquisition. The full scan range was 70–1,050 m/z with a resolution of 70,000, and the automatic gain control (AGC) target for MS acquisitions was set to 3e6 with a maximum ion injection time of 100 m. Top 3 precursors were selected for subsequent MSMS fragmentation with a maximum ion injection time of 50 m and resolution of 17,500, the AGC was 1e5. The stepped normalized collision energy was set to 20, 40 and 60 eV. ESI parameters were setting as: Sheath gas flow rate was 40, Aux gas flow rate was 10, positive-ion mode Spray voltage (|KV|) was 3.80, negative-ion mode Spray voltage (|KV|) was 3.2, Capillary temperature was 320 °C, Aux gas heater temperature was 350 °C. After importing the off-line data of mass spectrometry into compound discoverer 3.3 (Thermo Fisher Scientific, United States) software and analyzing the mass spectrometry data in combination with bmdb (BGI metabolome database), mzcloud database and chemspider online database, a data matrix containing information such as metabolite peak area and identification results will be obtained. After that, the table will be further analyzed and processed by Dr. TOM platform developed by BGI.

### Statistical analysis

Statistical analyses were performed using SPSS version 27.0. Normally distributed continuous variables were expressed as mean ± standard deviation (SD), while non-normally distributed variables were expressed as median (P25, P75). Categorical variables were presented as numbers (percentages) and analyzed using the chi-square test; Fisher’s exact test was used for binary variables or when expected frequencies were low. Kruskal-Wallis test was used for all data across multiple groups.

## Results

### Metabolite analysis of QFHZ decoction

**FIGURE 1:**
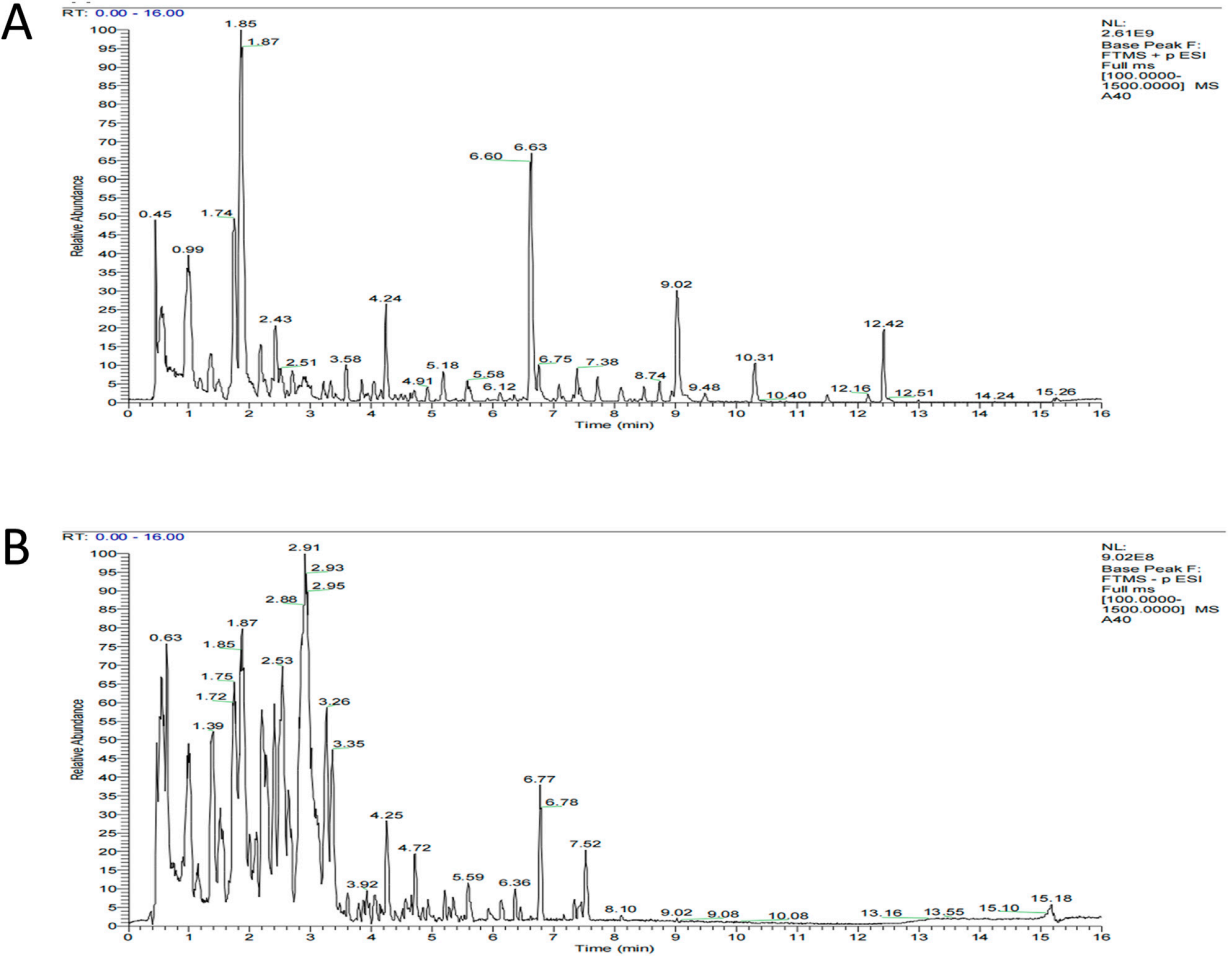
UHPLC- MS/MS analysis of QFHZ. **(A)** Total ion chromatography of QFHZ-treated rat serum in positive ion mode. **(B)** Total ion chromatography of QFHZ-treated rat serum in negative ion mode.

**FIGURE 2:**
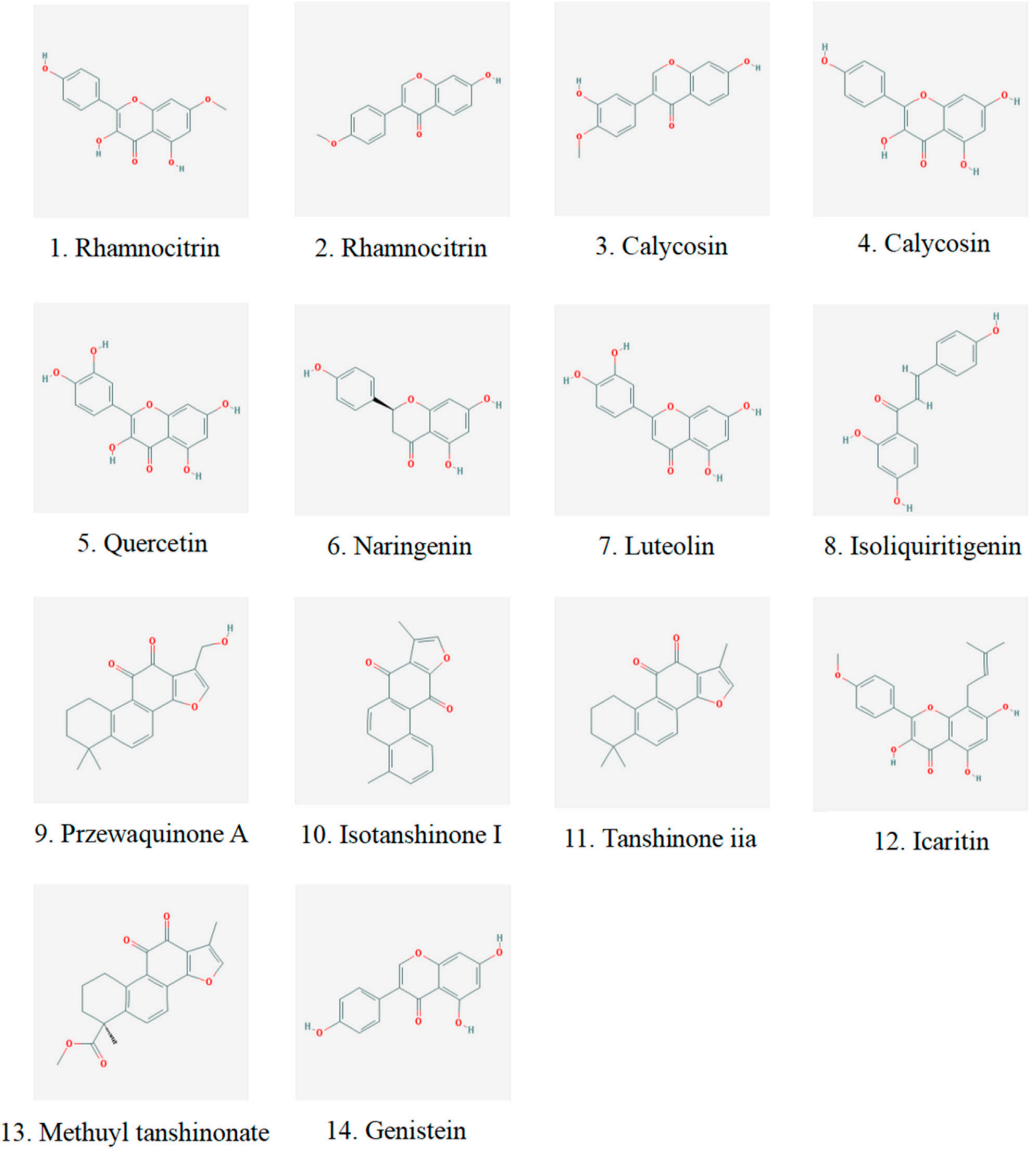
Chemical structure of components identified in QFHZ aqueous extract and rat serum.

We analyzed the metabolites of blank group serum, QFHZ drug-containing serum, and QFHZ water extracts using UPLC-Q-Exactive Orbitrap MS. A total of 5,969 metabolites were identified. The metabolites in QFHZ serum samples collected from rats were analyzed (Figure 1). 121 metabolites were filtered using the criteria of oral bioavailability (OB) ≥ 30% and drug-likeness (DL) ≥ 0.18. To further identify the main metabolites of QFHZ, the 121 metabolites were compared with the TCMSP database and literature reports. For more stringent metabolite identification, we exclusively considered metabolites detectable in both aqueous extracts and serum samples with a mass error of less than 5 ppm, ensuring they were both extractable and systemically available. Finally, we identified 14 major metabolites in QFHZ, with information provided in Table 2, The chemical structure of the 14 metabolites provided in Figure 2.

**TABLE 2:** The UHPLC- MS/MS data of the metabolites identified in the QFHZ.

NO.	m/z	Identity	Formula	Deviation (ppm)	t_R_/s	Ionization model	Category	MS/MS
1	301.070	Rhamnocitrin	C_20_H_20_O_6_	1.09297	205.27	M + H	Flavonoids	301.06831; 167.069235; 123.043824; 245.081003; 231.065245
2	269.080	Formononetin	C_16_H_12_O_4_	1.72433	311.50	[M + H]+	Isoflavone	269.08001; 254.057985; 213.089845; 237.052335; 253.04692
3	285.075	Calycosin	C_16_H_12_O_5_	0.67858	102.32	[M + H]+	Flavonoids	285.077375; 270.054047; 85.028522; 225.05314; 253.050274
4	285.040	Kaempferol	C_15_H_10_O_6_	1.74022	257.47	[M-H]-	Flavonoids	285.041817; 109.029622; 151.003945; 241.143044; 286.043996
5	303.050	Quercetin	C_15_H_10_O_7_	1.43152	205.75	[M + H]+	Flavonoids	303.04991; 137.023154; 229.050186; 257.045968; 153.018094
6	273.075	(±)-Naringenin	C_15_H_12_O_5_	1.60774	145.63	M + H	Flavonoids	273.076098; 153.018292; 147.043455; 171.027842; 119.049474
7	285.040	Luteolin	C_15_H_10_O_6_	0.45486	109.87	[M-H]-	Flavonoids	285.041851; 92.677817; 175.039057; 133.02865; 151.003962
8	257.081	Isoliquiritigenin	C_15_H_12_O_4_	1.53764	219.10	M + H	Flavonoids	257.079804; 137.023221; 147.044372; 211.074024; 239.06986
9	311.128	Przewaquinone A	C_19_H_18_O_4_	0.93380	140.91	[M + H]+	Quinones	137.059634; 251.107034; 311.127881; 279.101331; 219.080312
10	277.085	Isotanshinone I	C_18_H_12_O_3_	1.72134	537.93	[M + H]+	Quinones	277.086579; 249.089166; 193.09973; 178.077586; 254.530801
11	317.114	Tanshinone iia	C_19_H_18_O_3_	0.82734	618.36	[M + Na]+	Diterpene quinone	317.11468; 223.552224; 332.259341; 80.947874; 115.38001
12	369.133	Icaritin	C_21_H_20_O_6_	0.19304	427.66	[M + H]+	Flavonoids	313.068444; 369.132517; 135.044357; 298.048998; 92.657021
13	339.122	Methuyl tanshinonate	C_20_H_18_O_5_	0.05976	494.85	[M + H]+	Isopentenol lipids	261.090732; 279.101537; 233.094592; 339.119841; 205.101765
14	269.045	Genistein	C_15_H_10_O_5_	1.69755	451.61	[M-H]-	Flavonoids	269.045026; 225.056382; 241.050785; 291.691433; 111.881951

### Characteristics of study subjects

**FIGURE 3:**
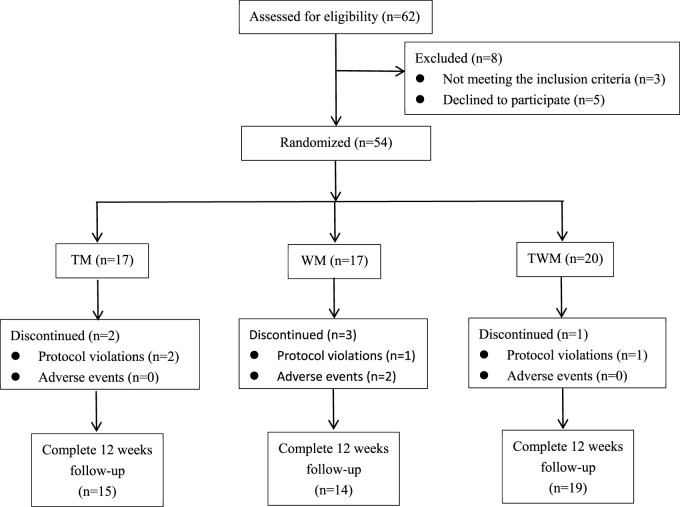
Participant flow through the trial.

We conducted a statistical analysis on all participates who completed the 12-week follow-up. Among the 62 participates, 54 participates were eligible to be enrolled in this trial based on inclusion criteria included from 4 July 2023 to 10 July 2024, a total of 48 completed the follow-up, with 15 in the TM group, 14 in the WM group, and 19 in the TWM group. Of the 6 participates who dropped out, 2 were from the TM group (both due to non-compliance with the treatment regimen), 3 were from the WM group (1 due to non-compliance, 1 due to hair loss and 1 due to dizziness), and 1 was from the WM group (due to non-compliance with the treatment regimen) (Figure 3). The baseline characteristics of all participants are presented in Table 3.

**TABLE 3:** Baseline characteristics of the study subjects.

Characteristic	TM (n = 17)	WM (n = 17)	TWM (n = 20)
Age (years)	40.88 ± 11.87	39.71 ± 11.80	37.60 ± 10.31
Sex, male, n (%)	17 (100%)	17 (100%)	20 (100%)
Height (m)	1.68 ± 0.07	1.72 ± 0.07	1.72 ± 0.06
Weight (kg)	73.79 ± 7.47	79.36 ± 12.60	76.65 ± 15.55
BMI (kg/m^2^)	26.12 ± 2.79	26.90 ± 2.97	25.89 ± 4.29
Time since gout diagnosis (years)	5.67 ± 2.83	5.85 ± 2.86	5.27 ± 2.66
CRP (mg/L)	4.40 (1.55, 6.95)	3.10 (0.60, 9.55)	4.50 (1.51, 6.98)
ESR (mm/h)	19.00 (14.00, 57.50)	27.00 (13.00, 46.50)	18.50 (12.00, 27.00)
UA (mg/dL)	9.03 ± 1.31	9.57 ± 1.75	9.30 ± 1.71
Cr (μmol/L)	88.59 ± 10.25	90.88 ± 9.83	92.95 ± 10.45
BUN (mg/dL)	5.13 ± 0.95	4.88 ± 1.19	4.65 ± 1.19
eGFR (ml/min/1.732 m^2^)	94.09 ± 14.81	93.28 ± 13.74	91.89 ± 15.51
Frequency of gout flare in 12 weeks before treatment	3.00 (1.50, 5.50)	1.00 (1.00, 3.00)	2.00 (1.00, 4.50)
Combined diseases, n (%)	6 (35.30)	6 (35.30)	10 (50.00)
Hypertension, n (%)	2 (11.76)	3 (17.65)	2 (10.00)
Hypertriglyceridemia, n (%)	4 (23.53)	5 (29.41)	9 (45.00)
Hypercholesterolemia, n (%)	3 (17.65)	2 (11.76)	2 (10.00)

UA, uric acid; CRP, C-reactive protein; ESR, erythrocyte sedimentation rate; Cr, creatinine; BUN, blood urea nitrogen; eGFR, estimated glomerular filtration rate.

### Evaluation of clinical efficacy

No significant differences were observed in changes in serum urate levels, urate-lowering target achievement rates, BUN, Cr or eGFR between the groups. An intergroup comparison revealed that TM can reduce the frequency of gout attacks compared to WM (*p =* 0.0006). Although combined therapy (TWM) reduced the frequency of gout flares, no significant difference was observed compared to Western medicine (WM) alone (Table 4).

**TABLE 4:** Clinical efficacy evaluation of participants with gout in the different group.

Outcome measure	TM (n = 15)	WM (n = 14)	TWM (n = 19)	*H/F*	*P value*
Percent change in serum urate concentration from baseline at final visit	−0.62 ± 1.48	−1.94 ± 1.96	−2.23 ± 2.18	2.65	0.082
∆Frequency of gout flare in comparision with 12 weeks before treatment	−3.00 (−4.00, −1.25)^a^	0.00 (−1.00, 1.00)	−1.00 (−2.00, 0.00)	12.56	0.002
∆BUN (mg/dL)	−0.05 ± 1.56	−0.13 ± 1.24	0.02 ± 1.15	0.03	0.986
∆eGFR (ml/min/1.732 m^2^)	8.77 ± 10.41	−2.18 ± 11.93	5.37 ± 11.25	3.33	0.046
∆Cr (μmol/L)	−8.25 ± 8.78	0.86 ± 11.21	−5.47 ± 10.00	2.87	0.068
Serum urate <6.0 mg/dL at week 12, n (%)	1 (6.67)	2 (14.29)	5 (26.32)	-	0.300
Decrease in frequency of gout flare after treatment, n (%)	13 (86.67)	6 (42.86)	14 (73.68)	-	0.045

^a^ *p* < 0.001 versus WM.

### Safety assessment

The adverse event rate in the TM was 17.65%, with all adverse reactions being itch. These symptoms generally appeared at the initiation of medication and resolved spontaneously within 1 week. In the WM, the adverse event rate was 35.29%, and abnormal renal function was the primary adverse reaction. The TWM group reported an adverse event rate of 15.00%, with thrombocytopenia identified as a noticeable adverse reaction. Except for itch, all adverse reactions in participants returned to normal after discontinuation of medication and appropriate medical treatment (Table 5).

**TABLE 5:** Self-reported side effects during 12-week treatment.

Adverse reaction	TM (n = 17)	WM (n = 17)	TWM (n = 20)	*P value*
Itch, n (%)	3 (17.65)	0 (0)	1 (5.00)	0.186
Abnormal liver function, n (%)	0 (0)	1 (5.88)	1 (5.00)	1.000
Abnormal renal function, n (%)	0 (0)	3 (17.65)	0 (0)	0.055
Hair loss, n (%)	0 (0)	1 (5.88)	0 (0)	0.637
Dizziness	0 (0)	1 (5.88)	0 (0)	0.637
Thrombocytopenia	0 (0)	0 (0)	1 (5.00)	1.000
Dropped due to adverse reactions	0 (0)	2 (11.76)	0 (0)	0.187
Any side effect(s)	3 (17.65)	6 (35.29)	3 (15.00)	0.351

### Overview of the proteomics results

**FIGURE 4:**
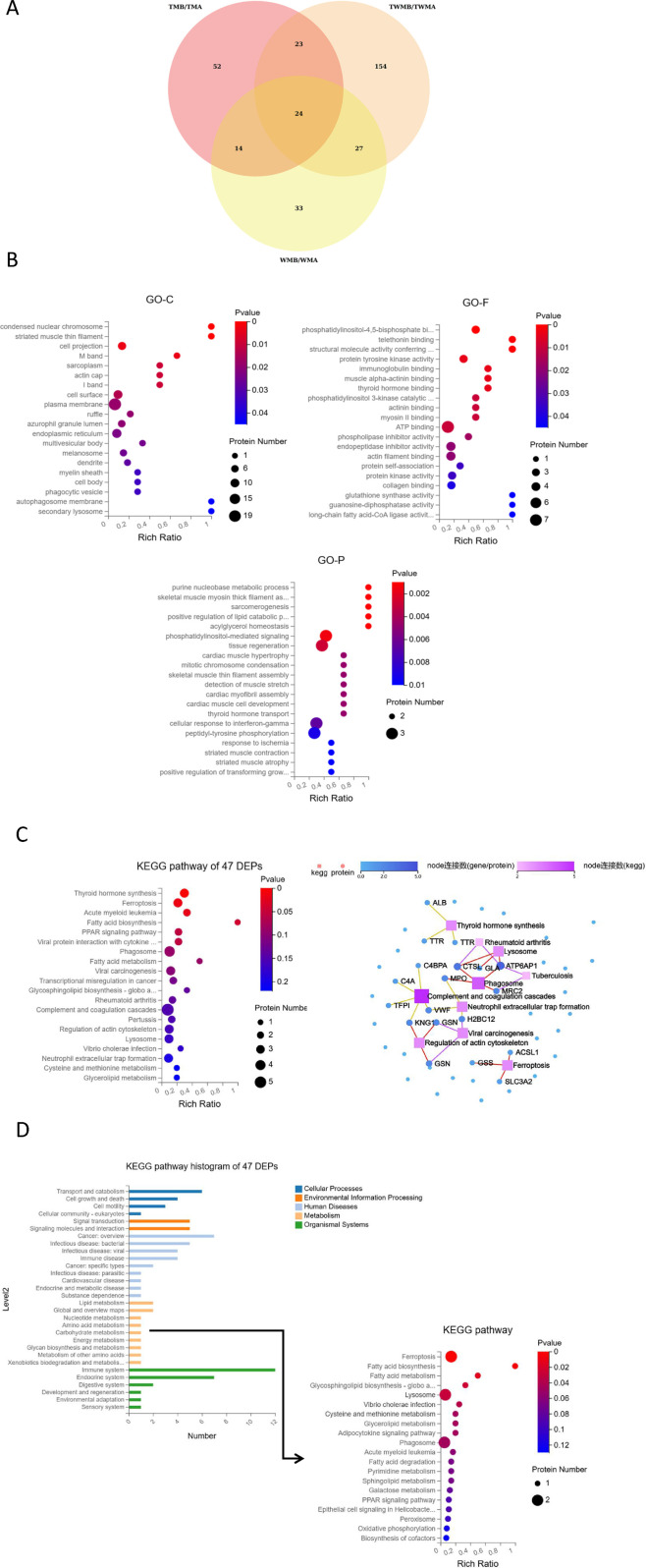
Overview of the proteomics results. **(A)** Venn diagram showed the overlapping proteins among the DEPs of TM, WM, and TWM before and after treatment (In group labeling, ‘A' represents pre-treatment while ‘B' represents post-treatment). **(B)** GO enrichment analysis of 47 DEPs shared by TM and TWM. **(C)** KEGG pathway and KEGG network analysis of 47 DEPs shared by TM and TWM. **(D)** KEGG pathway enrichment of six metabolism-related proteins from KEGG pathway histogram of 47 DEPs.

In proteomics analysis, we quantified 9,387 peptides and 1,630 proteins. Using criteria of fold change ≥1.2 or ≤0.83 and q-value <0.05, we identified 113, 98, and 228 DEPs in the TM, WM, and TWM groups, respectively. Among these, 47 DEPs were shared between TM and TWM, with 23 exclusive proteins (ALB, KNG1, IGHV4-59, ANGPTL3, GNG12, FGFBP2, ERP44, SUMF2, MIA, MMP15, AFM, ACSL1, hsa_circ_0042191, C4A, CTSL, CSF1R, MPO, hsa_circ_0051804, RP2, H2BC12, GLTPD2, FUOM) to these two groups, highlighting potential synergistic effects when combining QFHZ with standard Western medicine (WM) (Figure 4A). To explore QFHZ’s role, we focused on the 47 DEPs common to TM and TWM. GO enrichment analysis revealed these proteins were primarily localized in cell projections, condensed nuclear chromosomes, and plasma membranes, participating in critical biological processes such as purine nucleobase metabolism, positive regulation of lipid catabolism, and phosphatidylinositol-mediated signaling. Key molecular functions included phosphatidylinositol-4,5-bisphosphate binding, protein tyrosine kinase activity, and PI3K catalytic subunit binding, directly linking QFHZ to the PI3K-Akt signaling pathway, a critical regulator of cell survival and metabolism (Figure 4B). KEGG enrichment analysis revealed that Ferroptosis and Fatty acid biosynthesis are the major enrichment pathways. KEGG network interaction analysis highlighted 19 proteins with strong associations to key pathways, reinforcing the centrality of QFHZ’s action in pathways related to neutrophil extracellular trap formation, complement and coagulation cascades, lysosomes, phagosomes, and ferroptosis (Figure 4C; Supplementary Table S1). Consistent with the above results, KEGG pathway annotation revealed that six metabolism-related proteins (ATP6AP1, GSS, ACSL1, GLA, MPO, and ENTPD5) were primarily enriched in ferroptosis, lipid metabolism, and lysosomal pathways (Figure 4D).

### Overview of the metabolomics results

**FIGURE 5:**
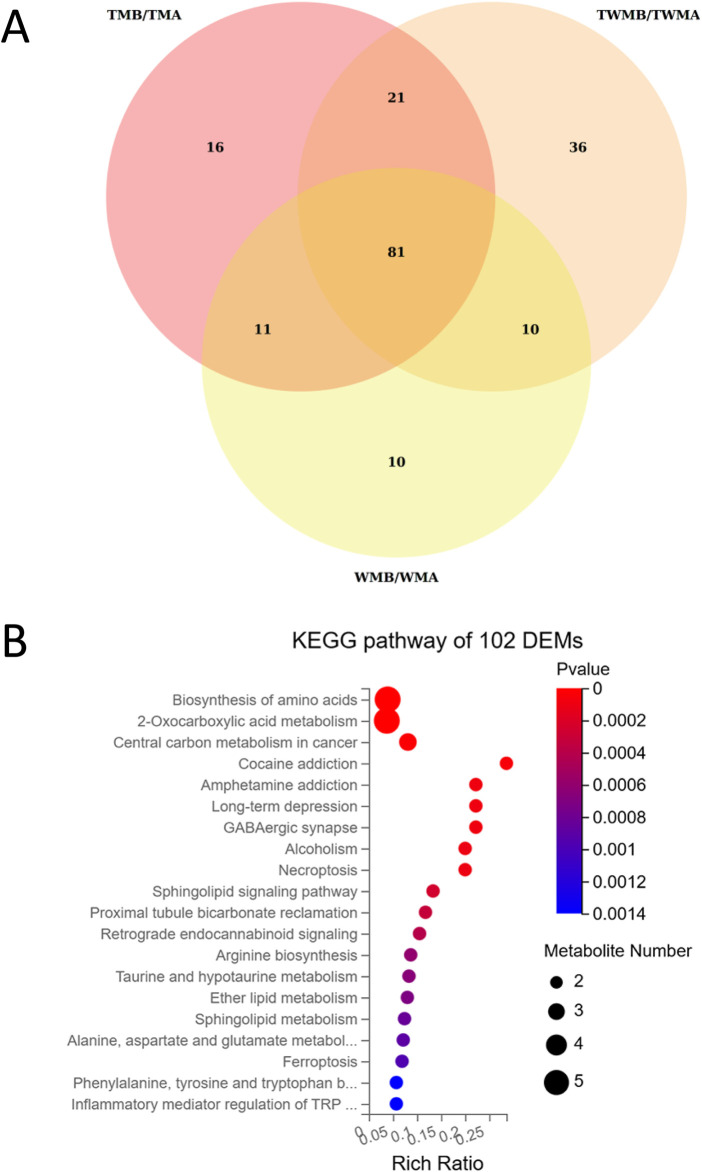
Overview of the metabolomics results. **(A)** Venn diagram showed the overlapping metabolites among the DEMs of TM, WM, and TWM before and after treatment. **(B)** KEGG pathway enrichment of 102 DEMs shared by TM and TWM.

To investigate the way by which QFHZ (TM group) prevents gout recurrence metabolomics analysis was conducted. DEMs were identified based on the following criteria: VIP ≥1, fold change ≥1.2 or ≤0.83, q-value <0.05, ppm range of −10 to 10, and a non-NA secondary spectrum score. DEMs were selected based on stringent criteria, revealing 129, 112, and 148 metabolites in the TM, WM, and TWM groups, respectively. According to Venn diagrams and KEGG pathway enrichment analysis, the DEMs before and after treatment in the three groups were highly similar (Figure 5A; Supplementary Figure S2). The main enriched pathways in all three groups included Biosynthesis of amino acids, Sphingolipid signaling pathway, 2-Oxocarboxylic acid metabolism, and Central carbon metabolism in cancer (Supplementary Figure S2). KEGG enrichment analysis of the overlapping metabolites between the TM and TWM groups identified key pathways, including 2-Oxocarboxylicacidmetabolism, Biosynthesis of amino acids, Central carbon metabolism in cancer and Ferroptosis (Figure 5B).

### Integrated proteomics and metabolomics analysis

**FIGURE 6:**
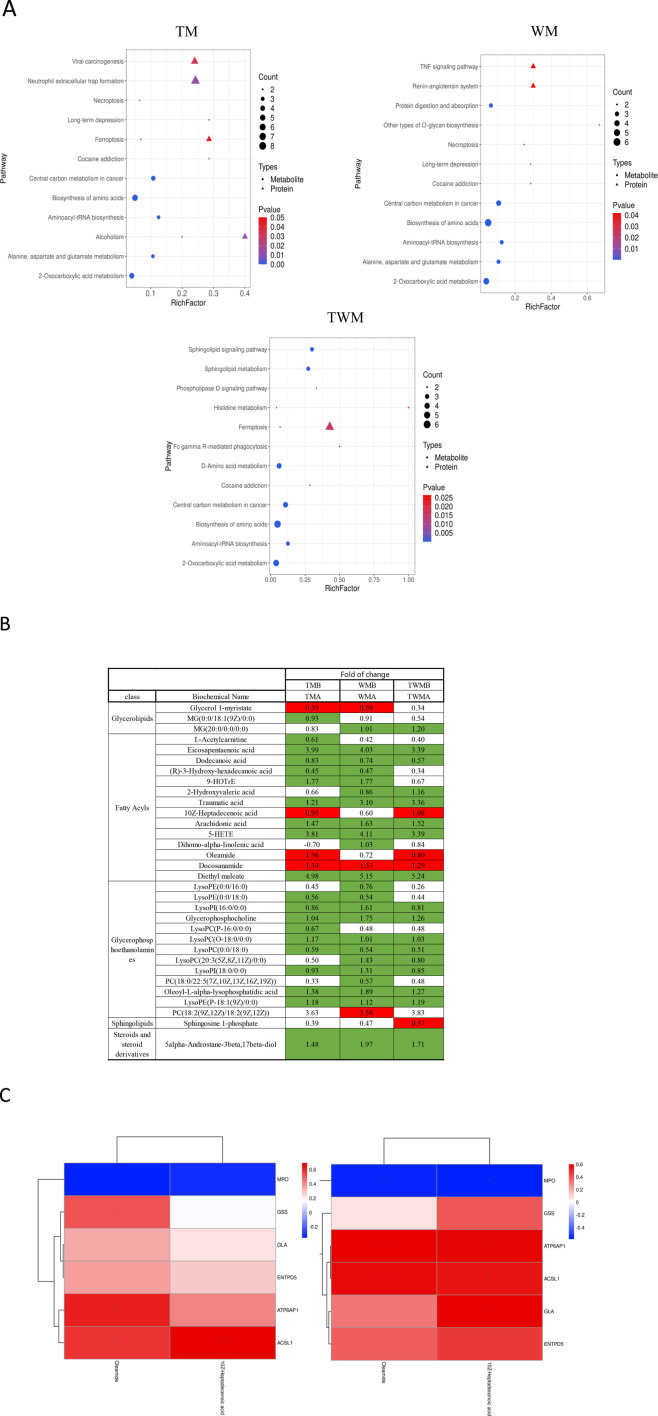
Integrated proteomics and metabolomics analysis. **(A)** Shared KEGG pathways with *p* < 0.05 between metabolites and proteins were explored for each group. The KEGG pathways, from left to right, represent the TM, WM, and TWM (In group labeling, ‘A' represents pre-treatment while ‘B' represents post-treatment). **(B)** Differential lipid metabolites of three groups. Green: indicates signifcant diference and Downregulation (*q*-value < 0.05, VIP ≥1, fold change ≤0.83), Red: indicates signifcant diference and upregulation (*q*-value < 0.05, VIP ≥1, fold change ≥1.2). **(C)** Correlation analysis between the proteins and metabolites. The left section corresponds to TM, the right section corresponds to TWM.*p < 0.05, **p < 0.01.

To explore the relationship between the metabolome and proteome of QFHZ, an integrated analysis was conducted, revealing distinct patterns across the TM, WM, and TWM groups. PCA score plots demonstrated clear separation of pre- and post-treatment samples among the three groups, highlighting distinct metabolic and proteomic profiles (Supplementary Figure S3). KEGG functional annotation revealed shared pathways between metabolites and proteins. Notably, the TM group exhibited overlaps in Alcoholism and Ferroptosis, while the TWM group shared Histidine metabolism and Ferroptosis between the two datasets. In contrast, no KEGG pathways were common between metabolomic and proteomic data in the WM group, suggesting limited integrative effects of Western medicine alone (Figure 6A). Considering ferroptosis is the major pathway shared by TM and TWM, detailed lipid metabolites were analysis. The TWM and WM groups exhibited notable differences primarily in Oleamide and 10Z-Heptadecenoic acid levels, potentially linked to against inflammation and ferroptosis, while the WM group lacked this increase and instead demonstrated a specific rise in PC(18:2 (9Z,12Z)/18:2 (9Z,12Z)), potentially linked to ferroptosis promotion (Figure 6B). These findings highlight the distinctive metabolic effects of QFHZ, setting it apart from WM.

To further investigate ferroptosis regulation, correlation analysis was conducted between the six metabolism-related proteins identified in the proteome and lipid metabolites including Oleamide and 10Z-Heptadecenoic acid in the TM and TWM groups. In the TWM group, ACSL1 was positively correlated with Oleamide (r = 0.57, *p* = 0.009) and 10Z-Heptadecenoic acid (r = 0.55, *p* = 0.011) (Figure 6C (left), Supplementary Table S2). Similar trends were observed in the TM group, with ACSL1 correlating positively with Oleamide (r = 0.58, *p* = 0.007) and 10Z-Heptadecenoic acid (r = 0.69, *p* < 0.001) (Figure 6C (right), Supplementary Table S3).

## Discussion

Gout, as a chronic inflammatory disease, requires long-term management. The treatment of gout requires a balance between meeting uric acid standards and preventing gout recurrence. Previous studies have shown that TCM can simultaneously address uric acid and inflammation (Fan et al., 2016). However, there is still a lack of clinical research on TCM treatment for gout, especially in preventing gout recurrence. QFHZ was established based on the “strengthening the body and nurturing vitality” principle of National Medical Master Li Jiren, aiming to treat gout by enhancing vital energy (Sun et al., 2023). Based on the results, we demonstrated that QFHZ may reduce the frequency of gout recurrence, which is consistent with our previous studies.

Our interim analysis results from 12-week trial provides preliminary evidence that TM group showed a statistically significant decrease in gout attacks compared to WM, while the TWM group exhibited a non-significant trend toward improvement. Notably, this effect appeared independent of serum urate reduction, as no significant intergroup differences were observed in uric acid levels. This dissociation suggests QFHZ’s benefits might involve non-urate-lowering pathways, which aligns with inflammatory modulation rather than direct uric acid control. However, among the 14 identified metabolite of QFHZ, 9 were flavonoids. Flavonoids are natural xanthine oxidase inhibitors, with specific metabolites including kaempferol, quercetin and luteolin having demonstrated XOD-inhibitory activity (Wang et al., 2020; Lee et al., 2017; De Souza et al., 2012). The absence of intergroup differences in urate-lowering efficacy may be attributed to shared pharmacological role between QFHZ and febuxostat. Nevertheless, the open-label design and reliance on self-reported flare frequency introduce potential bias, and—although complete study data are still needed—these limitations emphasize the necessity for larger, blinded confirmatory trials using objective outcome measures (e.g., ultrasound-confirmed synovitis).

The safety profile of QFHZ merits attention, particularly given its contrast with febuxostat therapy. While WM was associated with renal function abnormalities (17.65%) and higher overall adverse events (35.29%), QFHZ monotherapy reported only transient itch (17.65%). This suggests a potential tolerability advantage, especially for long-term prophylaxis in patients with comorbidities. Nevertheless, the small sample size preclude broad generalizations. Future trials should prioritize diverse populations and standardized concomitant therapies to clarify QFHZ’s risk-benefit profile.

In order to explore the role of QFHZ, a multi-omics approache was used. Proteomics and metabolics data analysis suggested that multi-target might be regulated by QFHZ. The initiation of acute gout is well-characterized by MSU crystal-induced NLRP3 inflammasome activation, leading to IL-1β release and NET-driven inflammation. In our study, reduced expression of MPO and vWF—key mediators of NETosis—was observed in the TM and TWM groups. Given the established roles of MPO in hypochlorous acid generation and vWF in NET stabilization (Li et al., 2025; Kim et al., 2023; Liu et al., 2023b), these data suggest that QFHZ might attenuate gout recurrence by indirectly influencing NET-related inflammation.

Complement activation exacerbates gouty inflammation via immune complex formation with MSU crystals (Fields et al., 1983). Our finding of decreased C4A (a complement cascade initiator) in TM/TWM groups aligns with reports that febuxostat may modulate complement proteins to reduce gout flares (Sanchez et al., 2024). But evidence for a direct link between the C4A downregulation and gout recurrence remains limited. Similarly, elevated lysosomal markers (GLA, ATP6AP1, and CTSL) across groups could imply lysosomal involvement, consistent with studies linking lysosomal acidification to IL-1β suppression (Duewell et al., 2010; Cobo et al., 2025).

Ferroptosis, marked by iron-dependent lipid peroxidation, has been implicated in gout progression (Wang et al., 2024; Chang et al., 2022; Du et al., 2023). Our data showed group-specific alterations in ferroptosis-associated metabolites (e.g., glutamate, arachidonic acid) and proteins (ACSL1, SLC3A2). Notably, the upregulation of the monounsaturated fatty acid 10Z-Heptadecenoic acid (anti-ferroptotic) and downregulation of pro-ferroptotic lipids (e.g., PC-PUFA2 in WM group) hint at a possible regulatory role of QFHZ in lipid peroxidation pathways. However, the paradoxical upregulation of ACSL1 (a putative pro-ferroptotic protein) alongside anti-ferroptotic lipids underscores the need to disentangle correlation from causation.

This study has several limitations. First, the sample size limits statistical power, necessitating validation through large-scale studies. Second, The study lacks a TCM syndrome evaluation scale, making it impossible to assess changes in TCM syndrome patterns. Third, the omics analysis was conducted in a small cohort, which may lead to reduced statistical power and potential overfitting in pathway enrichment. Fourth, the lack of direct evidence connecting these changes to QFHZ-specific mechanisms precludes definitive conclusions, therefore molecular mechanism validation is required. Notably, this study offers two key advances. First, Our phytochemical analysis of the QFHZ’s metabolites lends credibility to its hypothesized pharmacological mechanisms. Second, in a pioneering effort to integrate the QFHZ with omics analysis, our study systematically evaluated its role in preventing gout recurrence. This analysis revealed that QFHZ likely exerts its effects by modulating several biological processes that represent less explored therapeutic aspects, including complement activation, lysosomal function, and ferroptosis.

## Conclusion

Through this study, we found that QFHZ may have the potential to prevent acute gout attacks. Based on proteomics and metabolomics analysis, it is hypothesized that this effect may be related to QFHZ regulating multi-target. Future research should conduct more detailed mechanistic explorations using animal models and cellular models.

## Data Availability

The mass spectrometry proteomics data have been deposited to the ProteomeXchange Consortium (https://proteomecentral.proteomexchange.org) via the iProX partner repository [1,2] with the dataset identifier PXD069372.
