# Supplementary material for: Qifu Huazhuo formula for gout recurrence prevention: an interim analysis combining clinical outcomes with proteomic and metabolomic profiling
Source: Front Pharmacol. 2025 Oct 21;16:1642121. doi: 10.3389/fphar.2025.1642121 (PMC12582547; doi:10.3389/fphar.2025.1642121)
Supplement: Supplementary file 1 [file Supplementaryfile1.pdf]

Supplementary Materials

Table S1. 19 proteins of KEGG network interaction of 47 DEPs common among TM and TWM. 2
Table S2. Association between the six metabolism-related proteins and two lipid metabolites in TM. 4
Table S3. Association between the six metabolism-related proteins and two lipid metabolites in TWM. 4
Figure S1. Botanical drug quality report 5
Figure S2. KEGG pathway enrichment of DEMs in three groups. 38
Figure S3. PCA score plots of pre- and post-treatment samples among the three groups 39

Instruction: In group labeling, 'A' represents pre-treatment while 'B' represents post-treatment.

Table S1. 19 proteins of KEGG network interaction of 47 DEPs common among TM and TWM.

Protein ID	Symbol	Log2FC(TMB / TMA)	Pvalue(TMB / TMA)	Log2FC(TWMB / TWMA)	Pvalue(TWMB / TWMA)	Log2FC(WMB / WMA)	Pvalue(WMB / WMA)	ST RNA ID
tr|Q56G89|Q56G89_HUMAN	ALB	0.687416806	0.002764885	0.629222029	0.01195924	0.500327512	0.0535332	NM_000477
tr|B7Z9A0|B7Z9A0_HUMAN	GSN	0.762595278	0.003500026	1.460076439	0.00131347	0.714912289	0.002091156	NM_001353055
tr|B7Z2X4|B7Z2X4_HUMAN	GSN	1.057941814	3.313965E-4	1.117476362	9.305341E-6	0.998701991	0.005734551	NM_001353058
tr|B4DPP8|B4DPP8_HUMAN	KNG1	1.279993446	0.02398828	1.551491256	0.001526711	2.051956212	0.1117243	NM_000893
tr|A6XMH1|A6XMH1_HUMAN	TTR	1.333045505	3.190628E-9	1.723481616	1.671943E-4	1.269382845	8.151838E-5	NM_000371
tr|A0A087WT59|A0A087WT59_HUMAN	TTR	0.449254605	1.613848E-5	0.545256706	2.42035E-5	0.493109802	6.641584E-4	NM_000371
sp|Q9UBG0|MRC2_HUMAN	MRC2	0.779118624	9.875058E-4	1.091504711	0.002885876	0.454412298	0.0467775	NM_006039
sp|Q15904|VAS1_HUMAN	ATP6AP1	0.860170194	6.400114E-4	1.027671217	3.211559E-5	0.643971647	4.440218E-5	NM_001183
sp|P48637|GSHB_HUMAN	GSS	0.395859666	0.01751013	0.542517994	0.02016131	0.498657181	0.02225472	NM_001322495
sp|P33121|ACSL1_HUMAN	ACSL1	0.629428374	9.814816E-4	1.156910425	8.843466E-5	0.508867397	0.1258987	NM_001381878
sp|P10646|TFPI1_HUMAN	TFPI	0.399347168	0.004703277	0.820759915	2.724472E-4	0.324667335	0.02120102	XM_011511709

Table S1. (continued.)

<table>
<tr><th>Protein ID</th><th>Symbol</th><th>Log2FC(TMB / TMA)</th><th>Pvalue(TMB / TMA)</th><th>Log2FC(TWMB / TWMA)</th><th>Pvalue(TWMB / TWMA)</th><th>Log2FC(WMB / WMA)</th><th>Pvalue(WMB / WMA)</th><th>ST RNA ID</th></tr>
<tr><td>sp|P0C0L4|CO4A_HUMAN</td><td>C4A</td><td>-0.489124861</td><td>0.02512008</td><td>-0.630760113</td><td>0.02198406</td><td>-0.285408722</td><td>0.1909113</td><td>NM_007293</td></tr>
<tr><td>sp|P08195|4F2_HUMAN</td><td>SLC3A2</td><td>0.412238644</td><td>0.02862984</td><td>1.020683664</td><td>1.136624E-5</td><td>0.416778714</td><td>0.03098938</td><td>NM_002394</td></tr>
<tr><td>sp|P07711|CATL1_HUMAN</td><td>CTSL</td><td>0.381578528</td><td>0.04037016</td><td>0.499149045</td><td>0.006324214</td><td>0.307136714</td><td>0.08825771</td><td>NM_001257972</td></tr>
<tr><td>sp|P06280|AGAL_HUMAN</td><td>GLA</td><td>0.536552478</td><td>0.019233</td><td>0.965789752</td><td>1.535636E-4</td><td>0.500261455</td><td>4.483419E-4</td><td>NM_000169</td></tr>
<tr><td>sp|P05164|PERM_HUMAN</td><td>MPO</td><td>-0.42641039</td><td>0.03747819</td><td>-0.630869749</td><td>0.002492515</td><td>-0.231035433</td><td>0.4446155</td><td>NM_000250</td></tr>
<tr><td>sp|P04275|VWF_HUMAN</td><td>VWF</td><td>0.736961993</td><td>0.0072302332</td><td>1.304627319</td><td>0.001154561</td><td>1.165409562</td><td>0.005780568</td><td>NM_000552</td></tr>
<tr><td>sp|P04003|C4BPA_HUMAN</td><td>C4BPA</td><td>-0.330578686</td><td>0.004417267</td><td>-0.440331609</td><td>7.89151E-4</td><td>-0.349801589</td><td>0.001557469</td><td>XM_005273251</td></tr>
<tr><td>sp|O60814|H2B1K_HUMAN</td><td>H2BC12</td><td>-0.398546615</td><td>0.007822622</td><td>-0.723491479</td><td>0.02488434</td><td>0.131612151</td><td>0.6789505</td><td>NM_001312653</td></tr>
</table>

Table S2. Association between the six metabolism-related proteins and two lipid metabolites in TM.

	Oleamide	10Z-Heptadecenoic acid
ATP6AP1	**0.596992481**	**0.583458647**
GSS	0.07518797	0.398496241
ACSL1	**0.57078378**	**0.55271147**
GLA	0.327819549	**0.603007519**
MPO	**-0.569924812**	**-0.581954887**
ENTPD5	0.389473684	**0.470676692**

Bold: $p<0.05$

Table S3. Association between the six metabolism-related proteins and two lipid metabolites in TWM.

	Oleamide	10Z-Heptadecenoic acid
ATP6AP1	**0.627067669172932**	0.415037593984962
GSS	**0.520300751879699**	0.165413533834586
ACSL1	**0.57991730313953**	**0.69424600622281**
GLA	0.345864661654135	0.231578947368421
MPO	-0.353383459	-0.320300752
ENTPD5	0.360902255639098	0.282706766917293

Bold: $p<0.05$

Dioscorea hypoglauca

康美药业股份有限公司检测中心
检验报告

报告书编号：BM2023052400020　　　　KM-CX02205-01

检品名称	粉萆薢		产　地	江西	
检品来源	车间		包装规格	统装	
数　量	/		批　号	230502241	
取样量	210g		请检人	林少剑	
检验目的	入库检验		生产日期	/	
请检日期	2023 年 05 月 20 日		报告日期	2023 年 05 月 24 日	
检验依据	《中国药典》2020 年版一部				
检验项目	标准规定		检验结果		结论
【性状】*	应具有粉萆薢的性状特征		具有粉萆薢的性状特征		符合规定
【鉴别】					
显微鉴别*	应具有粉萆薢的显微特征		具有粉萆薢的显微特征		符合规定
薄层鉴别*	供试品色谱中，在与对照药材色谱相应的位置上，应显相同颜色的斑点或荧光斑点		供试品色谱中，在与对照药材色谱相应的位置上，显相同颜色的斑点或荧光斑点		符合规定
【检查】					
药屑、杂质	不得过 3%		0.3%		符合规定
水分	不得过 11.0%		10.1%		符合规定
总灰分*	不得过 3.0%		2.5%		符合规定
二氧化硫残留量*	不得过 150mg/kg		未检出		符合规定
【浸出物】*	不得少于 20.0%		24.6%		符合规定

注：二氧化硫残留量检出限：二氧化硫残留量小于 10mg/kg 视为未检出或 0mg/kg。

以下空白

检验结论	本品按《中国药典》2020 年版一部检验，结果符合规定。
备　注	带"*"的检测项目为引用中药材检验数据；报告以加盖检测中心检验报告专用章的纸质报告书为准

检验人：[signature]　　复核人：[signature]　　签发人：[signature]

Dioscorea hypoglauca

文件编码：KM-CX02205-02

康美药业股份有限公司检测中心
检验报告

报告编号：BM202505280026　　　　共1页，第1页

产品名称	粉萆薢	药材产地	江西吉安
装　　量	3g/5g/6g/10g/12g/15g/20g/25g/30g/[illegible]/60g/100g/250g/500g/600g/1kg/2kg/3kg/统装等		
规　　格	片	产品批号	250502701
取 样 量	200g	样品状态	完好
检验目的	入库检验	样品编号	20250524008
收样日期	2025年05月24日	报告/签发日期	2025年05月28日
实验活动日期	2025年05月24日至2025年05月28日		
检验依据	《中国药典》2020年版一部及四部		

检测项目	单位	评定指标	检测结果	检测方法	单项评定
【性状】*	/	具有粉萆薢的性状特征	符合规定	《中国药典》2020年版四部通则0212	符合
【鉴别】					
显微鉴别*	/	具有粉萆薢的显微特征	符合规定	《中国药典》2020年版四部通则2001	符合
薄层鉴别*	/	供试品色谱中，在与对照药材色谱相应的位置上，显相同颜色的斑点或荧光斑点	符合规定	《中国药典》2020年版四部通则0502	符合
【检查】					
药屑、杂质	%	≤3	0.2	《中国药典》2020年版四部通则2301	符合
水分	%	≤11.0	9.1	《中国药典》2020年版四部通则0832	符合
总灰分*	%	≤3.0	2.6	《中国药典》2020年版四部通则2302	符合
二氧化硫残留量*	mg/kg	≤150	未检出（检出限10）	《中国药典》2020年版四部通则2331	符合
【浸出物】*	%	≥20.0	27.9	《中国药典》2020年版四部通则2201	符合
检验结论	本品按《中国药典》2020年版一部及四部检验，结果符合规定。				
备　　注	带“*”的检测项目为引用中药材检验数据；报告以加盖检测中心检验报告专用章的纸质报告书为准				

---报告结束---

检验人：　　　　复核人：　　　　签发人：

Dioscorea hypoglauca

文件编码：KM-CX02205-02

康美药业股份有限公司检测中心
检验报告

报告编号：BM202410300021　　　　共1页，第1页

产品名称		粉萆薢	药材产地	江西吉安	
装　　量		3g/5g/6g/10g/12g/15g/20g/25g/30g/50g/60g/100g/250g/500g/600g/1kg/2kg/3kg/统装等			
规　　格		片	产品批号	241050251	
取 样 量		210g	样品状态	完好	
检验目的		入库检验	样品编号	20241027031	
收样日期		2024年10月27日	报告/签发日期	2024年10月30日	
实验活动日期		2024年10月27日至2024年10月30日			
检验依据		《中国药典》2020年版一部及四部			
**检测项目**	**单位**	**评定指标**	**检测结果**	**检测方法**	**单项评定**
【性状】*	/	具有粉萆薢的性状特征	符合规定	《中国药典》2020年版四部通则0212	符合
【鉴别】					
显微鉴别*	/	具有粉萆薢的显微特征	符合规定	《中国药典》2020年版四部通则2001	符合
薄层鉴别*	/	供试品色谱中，在与对照药材色谱相应的位置上，显相同颜色的斑点或荧光斑点	符合规定	《中国药典》2020年版四部通则0502	符合
【检查】					
药屑、杂质	%	≤3	0.2	《中国药典》2020年版四部通则2301	符合
水分*	%	≤11.0	8.7	《中国药典》2020年版四部通则0832	符合
总灰分*	%	≤3.0	2.4	《中国药典》2020年版四部通则2302	符合
二氧化硫残留量*	mg/kg	≤150	未检出（检出限10）	《中国药典》2020年版四部通则2331	符合
【浸出物】*	%	≥20.0	27.7	《中国药典》2020年版四部通则2201	符合
检验结论	本品按《中国药典》2020年版一部及四部检验，结果符合规定。				
备　　注	带"*"的检测项目为引用中药材检验数据；报告以加盖检测中心检验报告专用章的纸质报告书为准				

—报告结束—

检验人：[illegible]　　　复核人：[illegible]　　　签发人：[illegible]

Salvia miltiorrhiza

康美药业股份有限公司检测中心

检验报告

报告书编号：BM202306020046　　　　KM-CX02205-01

检品名称	丹参	产　　地	江苏
检品来源	车间	包装规格	统装
数　　量	/	批　　号	230550111
取 样 量	300g	请 检 人	赖浜豪
检验目的	入库检验	生产日期	/
请检日期	2023 年 05 月 18 日	报告日期	2023 年 06 月 02 日
检验依据	《中国药典》2020 年版一部		

检验项目	标准规定	检验结果	结论
【性状】	应具有丹参的性状特征	具有丹参的性状特征	符合规定
【鉴别】			
显微鉴别	应具有丹参的显微特征	具有丹参的显微特征	符合规定
薄层鉴别	供试品色谱中，在与对照药材色谱和对照品色谱相应的位置上，应显相同颜色的斑点或荧光斑点	供试品色谱中，在与对照药材色谱和对照品色谱相应的位置上，显相同颜色的斑点或荧光斑点	符合规定
【检查】			
药屑、杂质	不得过 3%	0.5%	符合规定
水分	不得过 13.0%	10.0%	符合规定
总灰分	不得过 10.0%	5.36%	符合规定
酸不溶性灰分	不得过 2.0%	1.0%	符合规定
重金属及有害元素	铅不得过 5mg/kg	0.1mg/kg	符合规定
	镉不得过 1mg/kg	0.02mg/kg	符合规定
	砷不得过 2mg/kg	0.04mg/kg	符合规定
	汞不得过 0.2mg/kg	0.002mg/kg	符合规定
	铜不得过 20mg/kg	4.3mg/kg	符合规定
二氧化硫残留量	不得过 150mg/kg	未检出	符合规定
【浸出物】			
水溶性浸出物	不得少于 35.0%	66.7%	符合规定
醇溶性浸出物	不得少于 11.0%	18.5%	符合规定
【含量测定】			
检验结论	本品按《中国药典》2020 年版一部检验，结果符合规定。		
备　　注	带"*"的检测项目为引用中药材检验数据；报告以加盖检测中心检验报告专用章的纸质报告书为准		

检验人：[illegible]　　复核人：[illegible]　　签发人：[illegible]

Salvia miltiorrhiza

文件编码：KM-CX02205-02

康美药业股份有限公司检测中心
检验报告

报告编号：BM202504240032 共1页，第1页

产品名称	丹参	药材产地	山东临沂
装　　量	3g/5g/6g/10g/12g/[illegible]/20g/25g/30g/50g/60g/100g/250g/500g/600g/1kg/2kg/3kg/统装等		
规　　格	片	产品批号	250402091
取 样 量	200g	样品状态	完好
检验目的	入库检验	样品编号	20250420046
收样日期	2025年04月20日	报告/签发日期	2025年04月24日
实验活动日期	2025年04月20日至2025年04月24日		
检验依据	《中国药典》2020年版一部及四部		

检测项目	单位	评定指标	检测结果	检测方法	单项评定
【性状】*	/	具有丹参的性状特征	符合规定	《中国药典》2020年版四部通则0212	符合
【鉴别】					
显微鉴别*	/	具有丹参的显微特征	符合规定	《中国药典》2020年版四部通则2001	符合
薄层鉴别*	/	供试品色谱中，在与对照药材色谱和对照品色谱相应的位置上，显相同颜色的斑点或荧光斑点	符合规定	《中国药典》2020年版四部通则0502	符合
【检查】					
药屑、杂质	%	≤3	0.4	《中国药典》2020年版四部通则2301	符合
水分	%	≤13.0	7.3	《中国药典》2020年版四部通则0832	符合
总灰分*	%	≤10.0	6.0	《中国药典》2020年版四部通则2302	符合
酸不溶性灰分*	%	≤2.0	1.5		符合
二氧化硫残留量*	mg/kg	≤150	未检出（检出限10）	《中国药典》2020年版四部通则2331	符合
【浸出物】					
水溶性浸出物*	%	≥35.0	60.7	《中国药典》2020年版四部通则2201	符合
醇溶性浸出物*	%	≥11.0	18.1		符合
检验结论	本品按《中国药典》2020年版一部及四部检验，结果符合规定。				
备　　注	带"*"的检测项目为引用中药材检验数据；报告以加盖检测中心检验报告专用章的纸质报告书为准				

---报告结束---

检验人：[illegible]　　复核人：[illegible]　　签发人：[illegible]

、

Salvia miltiorrhiza

文件编码：KM-CX02205-02

康美药业股份有限公司检测中心
检验报告

报告编号：BM202410230049　　　　共1页，第1页

产品名称	丹参		药材产地	山东临沂	
装　　量	3g/5g/6g/10g/12g/15g/20g/25g/30g/50g/[illegible]g/100g/250g/500g/600g/1kg/2kg/3kg/统装等				
规　　格	片		产品批号	241001261	
取 样 量	210g		样品状态	完好	
检验目的	入库检验		样品编号	20241021031	
收样日期	2024年10月21日		报告/签发日期	2024年10月23日	
实验活动日期	2024年10月22日至2024年10月23日				
检验依据	《中国药典》2020年版一部及四部				
检测项目	单位	评定指标	检测结果	检测方法	单项评定
【性状】*	/	具有丹参的性状特征	符合规定	《中国药典》2020年版四部通则0212	符合
【鉴别】					
显微鉴别*	/	具有丹参的显微特征	符合规定	《中国药典》2020年版四部通则2001	符合
薄层鉴别*	/	供试品色谱中，在与对照药材色谱和对照品色谱相应的位置上，显相同颜色的斑点或荧光斑点	符合规定	《中国药典》2020年版四部通则0502	符合
【检查】					
药屑、杂质	%	≤3	0.1	《中国药典》2020年版四部通则2301	符合
水分	%	≤13.0	6.5	《中国药典》2020年版四部通则0832	符合
总灰分*	%	≤10.0	5.51	《中国药典》2020年版四部通则2302	符合
酸不溶性灰分*	%	≤2.0	0.94		符合
二氧化硫残留量*	mg/kg	≤150	未检出（检出限10）	《中国药典》2020年版四部通则2331	符合
【浸出物】					
水溶性浸出物*	%	≥35.0	65.3	《中国药典》2020年版四部通则2201	符合
醇溶性浸出物*	%	≥11.0	18.9		符合
检验结论	本品按《中国药典》2020年版一部及四部检验，结果符合规定。				
备　　注	带"*"的检测项目为引用中药材检验数据；报告以加盖检测中心检验报告专用章的纸质报告书为准				

---报告结束---

检验人：　　　　复核人：　　　　签发人：

Astragalus mongholicus

康美药业股份有限公司检测中心
检验报告

报告书编号：BM202306200027　　　　　　　　　　KM-CX02205-01

检品名称	黄芪	产　　地	甘肃
检品来源	车间	包装规格	统装
数　　量	/	批　　号	230505461
取 样 量	210g	请 检 人	连乐江
检验目的	入库检验	生产日期	/
请检日期	2023 年 06 月 08 日	报告日期	2023 年 06 月 20 日
检验依据	《中国药典》2020 年版一部		

检验项目	标准规定	检验结果	结论
【性状】	应具有黄芪的性状特征	具有黄芪的性状特征	符合规定
【鉴别】			
显微鉴别*	应具有黄芪的显微特征	具有黄芪的显微特征	符合规定
薄层鉴别*	供试品色谱中，在与对照品色谱相应的位置上，日光下应显相同的棕褐色斑点；紫外光灯(365nm)下应显相同的橙黄色荧光斑点	供试品色谱中，在与对照品色谱相应的位置上，日光下显相同的棕褐色斑点；紫外光灯(365nm)下显相同的橙黄色荧光斑点	符合规定
	供试品色谱中，在与对照药材色谱相应的位置上，应显相同颜色的荧光主斑点	供试品色谱中，在与对照药材色谱相应的位置上，显相同颜色的荧光主斑点	符合规定
【检查】			
药屑、杂质	不得过 3%	0.2%	符合规定
水分	不得过 10.0%	7.3%	符合规定
总灰分*	不得过 5.0%	2.9%	符合规定
重金属及有害元素*	铅不得过 5mg/kg	0.2mg/kg	符合规定
	镉不得过 1mg/kg	0.04mg/kg	符合规定
	砷不得过 2mg/kg	0.3mg/kg	符合规定
	汞不得过 0.2mg/kg	未检出	符合规定
	铜不得过 20mg/kg	5.5mg/kg	符合规定
其他有机氯类农药残留量*	五氯硝基苯不得过 0.1mg/kg	未检出	符合规定
二氧化硫残留量*	不得过 150mg/kg	未检出	符合规定
检验结论	本品按《中国药典》2020 年版一部检验，结果符合规定。		
备　　注	带“*”的检测项目为引用中药材检验数据；报告以加盖检测中心检验报告专用章的纸质报告书为准		

检验人：[illegible]　　　　复核人：[illegible]　　　　签发人：[illegible]

Astragalus mongholicus

成都康美药业生产有限公司中心化验室
中药饮片检验报告

报告书编号：C20250612003　　　　REC-QP[illegible]-04

品　名	黄芪	药材产地	甘肃省定西市
检品来源	生产技术部	等　级	一等品
数　量	4938.2kg	批　号	250502961
收 样 量	300g	装　量	3g5g6g9g10g12g15g30g50g0.5kg1kg2kg
检验目的	入库检验	请检日期	2025.06.12
保 质 期	36 个月	生产日期	2025.05.28
检验日期	2025.06.12	报告日期	2025.06.12
检验依据	《中国药典》2020 年版第一增补本		

检验项目	标准规定	检验结果	结论
&【性状】	应具有黄芪的性状特征	具有黄芪的性状特征	符合规定
【鉴别】			
*显微鉴别	应具有黄芪的显微特征	具有黄芪的显微特征	符合规定
*薄层鉴别 1	供试品色谱中，在与对照品色谱相应的位置上，日光下应显相同的棕褐色斑点，紫外光(365nm) 下应显相同的橙黄色荧光斑点。	供试品色谱中，在与对照品色谱相应的位置上，日光下显相同的棕褐色斑点，紫外光(365nm) 下显相同的橙黄色荧光斑点。	符合规定
*薄层鉴别 2	供试品色谱中，在与对照药材色谱相应的位置上，应显相同颜色的荧光主斑点。	供试品色谱中，在与对照药材色谱相应的位置上，显相同颜色的荧光主斑点。	符合规定
【检查】			
&杂质	不得过 3.0%	0.2%	符合规定
&水分	不得过 10.0%	8.2%	符合规定
&总灰分	不得过 5.0%	2.6%	符合规定
*重金属及有害元素	铅不得过 5mg/kg；镉不得过 1mg/kg；砷不得过 2mg/kg；汞不得过 0.2mg/kg；铜不得过 20mg/kg	铅：0.6mg/kg；　镉：0mg/kg；砷：0.8mg/kg；　汞：0.1mg/kg；铜：7.0mg/kg	符合规定
*其他有机氯类农药残留量	五氯硝基苯不得过 0.1mg/kg	0mg/kg	符合规定
*二氧化硫残留量	不得过 150mg/kg	未检出（检出限 3mg/kg）	符合规定
&【浸出物】	不得少于 17.0%	38.4%	符合规定
&【含量测定】	本品按干燥品计算，含黄芪甲苷含量不得少于 0.080%	0.104%	符合规定
	含毛蕊异黄酮葡萄糖苷含量不得少于 0.020%	0.052%	符合规定
【装量差异】	应符合规定	符合规定	符合规定

——报告结束——

检验结论	本品按《中国药典》2020 年版第一增补本检验，结果符合规定。
备　注	带“*”的项目为引用该批次使用的中药材检验数据；带“&”的项目为引用的该批次待包装产品的检验数据；检验报告未加盖本实验室“检验专用章”无效。

检验人：　　　　复核人：　　　　授权签字人：

Astragalus mongholicus

文件编码：KM-CX02205-02

康美药业股份有限公司检测中心
检验报告

报告编号：BM202404170040 共2页，第1页

检品名称	黄芪	产地	甘肃
检品来源	车间	规格	统装
请检人	赖浜斐	批号	240350391
取样量	300g	样品状态	完好
检验目的	入库检验	样品编号	20240410042
收样日期	2024年04月10日	报告/签发日期	2024年04月17日
实验活动日期	2024年04月11日至2024年04月17日		
检验依据	《中国药典》2020年版一部及四部		

检测项目	单位	评定指标	检测结果	检测方法	单项评定
【性状】	/	具有黄芪的性状特征	符合规定	《中国药典》2020年版四部通则0212	符合
【鉴别】					
显微鉴别*	/	具有黄芪的显微特征	符合规定	《中国药典》2020年版四部通则2001	符合
薄层鉴别*	/	供试品色谱中，在与对照品色谱相应的位置上，日光下显相同的棕褐色斑点；紫外光(365nm)下显相同的橙黄色荧光斑点	符合规定	《中国药典》2020年版四部通则0502	符合
	/	供试品色谱中，在与对照药材色谱相应的位置上，显相同颜色的荧光主斑点	符合规定		符合
【检查】					
药屑、杂质	%	≤3	0.3	《中国药典》2020年版四部通则2301	符合
水分	%	≤10.0	8.7	《中国药典》2020年版四部通则0832	符合
总灰分*	%	≤5.0	3.1	《中国药典》2020年版四部通则2302	符合
重金属及有害元素*	mg/kg	铅≤5	0.1（检出限15ppt）	《中国药典》2020年版四部通则2321	符合
	mg/kg	镉≤1	0.03（检出限5ppt）		符合
	mg/kg	砷≤2	0.1（检出限7ppt）		符合
	mg/kg	汞≤0.2	0.002（检出限20ppt）		符合
	mg/kg	铜≤20	4.3（检出限2ppt）		符合
其他有机氯类农药残留量*	mg/kg	五氯硝基苯≤0.1	未检出（检出限以α-六六六计≤0.000006）	《中国药典》2020年版四部通则2341	符合
二氧化硫残留量*	mg/kg	≤150	未检出（检出限10）	《中国药典》2020年版四部通则2331	符合
检验结论	本品按《中国药典》2020年版一部及四部检验，结果符合规定。				
备注	带“*”的检测项目为引用中药材检验数据；报告以加盖检测中心检验报告专用章的纸质报告书为准				

检验人：[illegible] 复核人：[illegible] 签发人：[illegible]

Smilax glabra

康美药业股份有限公司检测中心

检验报告

报告书编号：BM202305140028　　　　　　KM-CX02205-01

检品名称	土茯苓	产　　地	广西
检品来源	车间	包装规格	统装
数　　量	/	批　　号	230500711
取 样 量	210g	请 检 人	黄宗贵
检验目的	入库检验	生产日期	/
请检日期	2023 年 05 月 11 日	报告日期	2023 年 05 月 14 日
检验依据	《中国药典》2020 年版一部		

检验项目	标准规定	检验结果	结论
【性状】*	应具有土茯苓的性状特征	具有土茯苓的性状特征	符合规定
【鉴别】			
显微鉴别*	应具有土茯苓的显微特征	具有土茯苓的显微特征	符合规定
薄层鉴别*	供试品色谱中，在与对照品色谱相应的位置上，应显相同颜色的荧光斑点	供试品色谱中，在与对照品色谱相应的位置上，显相同颜色的荧光斑点	符合规定
【检查】			
药屑、杂质	不得过 3%	0.1%	符合规定
水分	不得过 15.0%	8.4%	符合规定
总灰分*	不得过 5.0%	0.99%	符合规定
二氧化硫残留量*	不得过 150mg/kg	未检出	符合规定
【浸出物】*	不得少于 10.0%	28.7%	符合规定
【含量测定】*	本品按干燥品计算，含落新妇苷（$C_{21}H_{22}O_{11}$）不得少于 0.45%	2.9%	符合规定

注：二氧化硫残留量检出限：二氧化硫残留量小于 10mg/kg 视为未检出或 0mg/kg。

以下空白

检验结论	本品按《中国药典》2020 年版一部检验，结果符合规定。
备　　注	带“*”的检测项目为引用中药材检验数据；报告以加盖检测中心检验报告专用章的纸质报告书为准

检验人：[illegible]　　复核人：[illegible]　　签发人：[illegible]

Smilax glabra

文件编码：KM-CX02205-02

康美药业股份有限公司检测中心
检验报告

报告编号：BM202506070005

共1页，第1页

产品名称	土茯苓	药材产地	云南红河
装 量	3g/5g/6g/10g/12g/15g/20g/25g/30g/50g/60g/100g/250g/500g/600g/1kg/2kg/3kg/统装等		
规 格	片	产品批号	250504571
取 样 量	200g	样品状态	完好
检验目的	入库检验	样品编号	20250603003
收样日期	2025年06月03日	报告/签发日期	2025年06月07日
实验活动日期	2025年06月03日至2025年06月07日		
检验依据	《中国药典》2020年版一部及四部		

检测项目	单位	评定指标	检测结果	检测方法	单项评定
【性状】	/	具有土茯苓的性状特征	符合规定	《中国药典》2020年版四部通则0212	符合
【鉴别】					
显微鉴别*	/	具有土茯苓的显微特征	符合规定	《中国药典》2020年版四部通则2001	符合
薄层鉴别*	/	供试品色谱中，在与对照品色谱相应的位置上，显相同颜色的荧光斑点	符合规定	《中国药典》2020年版四部通则0502	符合
【检查】					
药屑、杂质	%	≤3	0.2	《中国药典》2020年版四部通则2301	符合
水分	%	≤15.0	8.9	《中国药典》2020年版四部通则0832	符合
总灰分*	%	≤5.0	0.9	《中国药典》2020年版四部通则2302	符合
二氧化硫残留量*	mg/kg	≤150	未检出（检出限10）	《中国药典》2020年版四部通则2331	符合
【浸出物】*	%	≥10.0	30.5	《中国药典》2020年版四部通则2201	符合
【含量测定】					
落新妇苷*	%	≥0.45	3.67	《中国药典》2020年版四部通则0512	符合
检验结论	本品按《中国药典》2020年版一部及四部检验，结果符合规定。				
备 注	带“*”的检测项目为引用中药材检验数据，报告以加盖检测中心检验报告专用章的纸质报告书为准				

—报告结束—

检验人：　　复核人：　　签发人：

Smilax glabra

文件编码：KM-CX02205-02

康美药业股份有限公司检测中心
检验报告

报告编号：BM202410120060

共1页，第1页

产品名称	土茯苓	药材产地	云南红河
装　　量	3g/5g/6g/10g/12g/15g/20g/25g/30g/50g/60g/100g/250g/500g/600g/1kg/2kg/3kg/统装等		
规　　格	片	产品批号	241050031
取 样 量	210g	样品状态	完好
检验目的	入库检验	样品编号	20241011010
收样日期	2024年10月11日	报告/签发日期	2024年10月12日
实验活动日期	2024年10月11日至2024年10月12日		
检验依据	《中国药典》2020年版一部及四部		

检测项目	单位	评定指标	检测结果	检测方法	单项评定
【性状】*	/	具有土茯苓的性状特征	符合规定	《中国药典》2020年版四部通则0212	符合
【鉴别】					
显微鉴别*	/	具有土茯苓的显微特征	符合规定	《中国药典》2020年版四部通则2001	符合
薄层鉴别*	/	供试品色谱中，在与对照品色谱相应的位置上，显相同颜色的荧光斑点	符合规定	《中国药典》2020年版四部通则0502	符合
【检查】					
药屑、杂质	%	≤3	0.3	《中国药典》2020年版四部通则2301	符合
水分*	%	≤15.0	8.3	《中国药典》2020年版四部通则0832	符合
总灰分*	%	≤5.0	1.1	《中国药典》2020年版四部通则2302	符合
二氧化硫残留量*	mg/kg	≤150	未检出（检出限10）	《中国药典》2020年版四部通则2331	符合
【浸出物】*	%	≥10.0	31.8	《中国药典》2020年版四部通则2201	符合
【含量测定】					
落新妇苷*	%	≥0.45	2.9	《中国药典》2020年版四部通则0512	符合
检验结论	本品按《中国药典》2020年版一部及四部检验，结果符合规定。				
备　　注	带“*”的检测项目为引用中药材检验数据；报告以加盖检测中心检验报告专用章的纸质报告书为准				

---报告结束---

检验人：[illegible]　　复核人：[illegible]　　签发人：[illegible]

Cornus officinalis

康美药业股份有限公司检测中心
检验报告

报告书编号：BM202306070006　　　　KM-CX02205-01

检品名称	山萸肉	产　　地	河南
检品来源	车间	包装规格	统装
数　　量	/	批　　号	230504121
取 样 量	210g	请 检 人	林少剑
检验目的	入库检验	生产日期	/
请检日期	2023 年 06 月 01 日	报告日期	2023 年 06 月 07 日
检验依据	《中国药典》2020 年版一部		

检验项目	标准规定	检验结果	结论
【性状】	应具有山萸肉的性状特征	具有山萸肉的性状特征	符合规定
【鉴别】			
显微鉴别*	应具有山茱萸的显微特征	具有山茱萸的显微特征	符合规定
薄层鉴别*	供试品色谱中，在与对照品色谱相应的位置上，应显相同的紫红色斑点；置紫外光灯（365nm）下检视，应显相同的橙黄色荧光斑点	供试品色谱中，在与对照品色谱相应的位置上，显相同的紫红色斑点；置紫外光灯（365nm）下检视，显相同的橙黄色荧光斑点	符合规定
	供试品色谱中，在与对照品色谱相应的位置上，应显相同颜色的荧光斑点	供试品色谱中，在与对照品色谱相应的位置上，显相同颜色的荧光斑点	符合规定
【检查】			
药屑、杂质	不得过 3%	0.4%	符合规定
水分	不得过 16.0%	9.6%	符合规定
总灰分*	不得过 6.0%	4.1%	符合规定
二氧化硫残留量*	不得过 150mg/kg	未检出	符合规定
【浸出物】*	不得少于 50.0%	65.5%	符合规定
【含量测定】*	本品按干燥品计算，含莫诺苷（$C_{17}H_{26}O_{11}$）和马钱苷（$C_{17}H_{26}O_{10}$）的总量不得少于 1.2%	2.7%	符合规定

注：二氧化硫残留量检出限：二氧化硫残留量小于 10mg/kg 视为未检出或 0mg/kg。

以下空白

检验结论	本品按《中国药典》2020 年版一部检验，结果符合规定。
备　　注	带“*”的检测项目为引用中药材检验数据；报告以加盖检测中心检验报告专用章的纸质报告书为准

检验人：[illegible]　　复核人：[illegible]　　签发人：[illegible]

Cornus officinalis

文件编码：KM-CX02205-02

康美药业股份有限公司检测中心
检验报告

报告编号：BM202504230017　　　　共1页，第1页

产品名称		山萸肉	药材产地	河南西峡	
装　　量		3g/5g/6g/10g/12g/1[illegible]g/20g/25g/30g/50g/[illegible]0g/100g/250g/500g/600g/1kg/2kg/3kg/统装等			
规　　格		/	产品批号	250402521	
取 样 量		200g	样品状态	完好	
检验目的		入库检验	样品编号	20250418052	
收样日期		2025年04月18日	报告/签发日期	2025年04月23日	
实验活动日期		2025年04月18日至2025年04月23日			
检验依据		《中国药典》2020年版一部及四部			
**检测项目**	**单位**	**评定指标**	**检测结果**	**检测方法**	**单项评定**
【性状】*	/	具有山萸肉的性状特征	符合规定	《中国药典》2020年版四部通则0212	符合
【鉴别】					
显微鉴别*	/	具有山茱萸的显微特征	符合规定	《中国药典》2020年版四部通则2001	符合
薄层鉴别*	/	供试品色谱中，在与对照品色谱相应的位置上，显相同的紫红色斑点；置紫外光灯（365nm）下检视，显相同的橙黄色荧光斑点	符合规定	《中国药典》2020年版四部通则0502	符合
	/	供试品色谱中，在与对照品色谱相应的位置上，显相同颜色的荧光斑点	符合规定		符合
【检查】					
药屑、杂质	%	≤3	0.4	《中国药典》2020年版四部通则2301	符合
水分	%	≤16.0	10.3	《中国药典》2020年版四部通则0832	符合
总灰分*	%	≤6.0	4.3	《中国药典》2020年版四部通则2302	符合
二氧化硫残留量*	mg/kg	≤150	未检出（检出限10）	《中国药典》2020年版四部通则2331	符合
【含量测定】					
莫诺苷和马钱苷的总量*	%	≥1.2	2.9	《中国药典》2020年版四部通则0512	符合
检验结论	本品按《中国药典》2020年版一部及四部检验，结果符合规定。				
备　　注	带“*”的检测项目为引用中药材检验数据；报告以加盖检测中心检验报告专用章的纸质报告书为准				

---报告结束---

检验人：[illegible]　　复核人：[illegible]　　签发人：[illegible]

Cornus officinalis

文件编码：KM-CX02205-02

康美药业股份有限公司检测中心
检验报告

报告编号：BM202501050031　　　　共1页，第1页

产品名称	山萸肉	药材产地	河南西峡
装　　量	3g/5g/6g/10g/12g/15g/20g/25g/30g/50g/60g/100g/250g/500g/600g/1kg/2kg/3kg/统装等		
规　　格	/	产品批号	241204511
取 样 量	200g	样品状态	完好
检验目的	入库检验	样品编号	20250102059
收样日期	2025年01月02日	报告/签发日期	2025年01月05日
实验活动日期	2025年01月02日至2025年01月05日		
检验依据	《中国药典》2020年版一部及四部		

检测项目	单位	评定指标	检测结果	检测方法	单项评定
【性状】*	/	具有山萸肉的性状特征	符合规定	《中国药典》2020年版四部通则0212	符合
【鉴别】					
显微鉴别*	/	具有山茱萸的显微特征	符合规定	《中国药典》2020年版四部通则2001	符合
薄层鉴别*	/	供试品色谱中，在与对照品色谱相应的位置上，显相同的紫红色斑点；置紫外光灯（365nm）下检视，显相同的橙黄色荧光斑点	符合规定	《中国药典》2020年版四部通则0502	符合
	/	供试品色谱中，在与对照品色谱相应的位置上，显相同颜色的荧光斑点	符合规定		符合
【检查】					
药屑、杂质	%	≤3	0.3	《中国药典》2020年版四部通则2301	符合
水分	%	≤16.0	9.7	《中国药典》2020年版四部通则0832	符合
总灰分*	%	≤6.0	4.5	《中国药典》2020年版四部通则2302	符合
二氧化硫残留量*	mg/kg	≤150	未检出（检出限10）	《中国药典》2020年版四部通则2331	符合
【含量测定】					
莫诺苷和马钱苷的总量*	%	≥1.2	2.7	《中国药典》2020年版四部通则0512	符合
检验结论	本品按《中国药典》2020年版一部及四部检验，结果符合规定。				
备　　注	带“*”的检测项目为引用中药材检验数据；报告以加盖检测中心检验报告专用章的纸质报告书为准				

---报告结束---

检验人：[illegible]　　　复核人：[illegible]　　　签发人：[illegible]

康美药业股份有限公司检测中心
检验报告

报告书编号：BM202306060035 KM-CX02205-01

检品名称	盐杜仲	产　　地	湖北
检品来源	车间	包装规格	统装
数　　量	/	批　　号	230502141
取 样 量	210g	请 检 人	林财逢
检验目的	入库检验	生产日期	/
请检日期	2023 年 05 月 26 日	报告日期	2023 年 06 月 06 日
检验依据	《中国药典》2020 年版一部		

检验项目	标准规定	检验结果	结论
【性状】	应具有盐杜仲的性状特征	具有盐杜仲的性状特征	符合规定
【鉴别】			
显微鉴别*	应具有杜仲的显微特征	具有杜仲的显微特征	符合规定
理化鉴别*	应呈正反应	呈正反应	符合规定
【检查】			
药屑、杂质	不得过 3%	0.4%	符合规定
水分	不得过 13.0%	5.8%	符合规定
总灰分	不得过 10.0%	5.96%	符合规定
二氧化硫残留量*	不得过 150mg/kg	未检出	符合规定
【浸出物】	不得少于 12.0%	16.6%	符合规定
【含量测定】	本品含松脂醇二葡萄糖苷 ($C_{32}H_{42}O_{16}$) 不得少于 0.10%	0.22%	符合规定

注：二氧化硫残留量检出限：二氧化硫残留量小于 10mg/kg 视为未检出或 0mg/kg。

以下空白

检验结论	本品按《中国药典》2020 年版一部检验，结果符合规定。
备　　注	带“*”的检测项目为引用中药材检验数据；报告以加盖检测中心检验报告专用章的纸质报告书为准

检验人：[illegible]　　复核人：[illegible]　　签发人：[illegible]

Eucommia ulmoides

文件编码：KM-CX02205-02

康美药业股份有限公司检测中心
检验报告

报告编号：BM202505060014　　　　共1页，第1页

产品名称		盐杜仲	药材产地	四川广元	
装　　量		3g/5g/6g/10g/12g/15g/20g/25g/30g/50g/60g/100g/250g/500g/600g/1kg/2kg/3kg/统装等			
规　　格		块	产品批号	250403461	
取 样 量		200g	样品状态	完好	
检验目的		入库检验	样品编号	20250430035	
收样日期		2025年04月30日	报告/签发日期	2025年05月06日	
实验活动日期		2025年04月30日至2025年05月06日			
检验依据		《中国药典》2020年版一部及四部			
检测项目	单位	评定指标	检测结果	检测方法	单项评定
【性状】	/	具有盐杜仲的性状特征	符合规定	《中国药典》2020年版四部通则0212	符合
【鉴别】					
显微鉴别*	/	具有杜仲的显微特征	符合规定	《中国药典》2020年版四部通则2001	符合
理化鉴别*	/	呈正反应	符合规定	《中国药典》2020年版一部（杜仲）理化鉴别项下	符合
【检查】					
药屑、杂质	%	≤3	0.1	《中国药典》2020年版四部通则2301	符合
水分	%	≤13.0	4.0	《中国药典》2020年版四部通则0832	符合
总灰分	%	≤10.0	5.8	《中国药典》2020年版四部通则2302	符合
二氧化硫残留量*	mg/kg	≤150	未检出（检出限10）	《中国药典》2020年版四部通则2331	符合
【浸出物】	%	≥12.0	18.9	《中国药典》2020年版四部通则2201	符合
【含量测定】					
松脂醇二葡萄糖苷	%	≥0.10	0.22	《中国药典》2020年版四部通则0512	符合
检验结论	本品按《中国药典》2020年版一部及四部检验，结果符合规定。				
备　　注	带"*"的检测项目为引用中药材检验数据；报告以加盖检测中心检验报告专用章的纸质报告书为准				

---报告结束---

检验人：[illegible]　　复核人：[illegible]　　签发人：[illegible]

Eucommia ulmoides

文件编码：KM-CX02205-02

康美药业股份有限公司检测中心
检验报告

报告编号：BM202411210008

共1页，第1页

产品名称	盐杜仲		药材产地	四川广元	
装　　量	3g/5g/6g/10g/12g/15g/20g/25g/30g/50g/60g/100g/250g/500g/600g/1kg/2kg/3kg/统装等				
规　　格	块		产品批号	241004211	
取 样 量	200g		样品状态	完好	
检验目的	入库检验		样品编号	20241115019	
收样日期	2024年11月15日		报告/签发日期	2024年11月21日	
实验活动日期	2024年11月15日至2024年11月21日				
检验依据	《中国药典》2020年版一部及四部				
检测项目	单位	评定指标	检测结果	检测方法	单项评定
【性状】	/	具有盐杜仲的性状特征	符合规定	《中国药典》2020年版四部通则0212	符合
【鉴别】					
显微鉴别*	/	具有杜仲的显微特征	符合规定	《中国药典》2020年版四部通则2001	符合
理化鉴别*	/	呈正反应	符合规定	《中国药典》2020年版一部（杜仲）理化鉴别项下	符合
【检查】					
药屑、杂质	%	≤3	0.4	《中国药典》2020年版四部通则2301	符合
水分	%	≤13.0	3.6	《中国药典》2020年版四部通则0832	符合
总灰分	%	≤10.0	5.91	《中国药典》2020年版四部通则2302	符合
二氧化硫残留量*	mg/kg	≤150	未检出（检出限10）	《中国药典》2020年版四部通则2331	符合
【浸出物】	%	≥12.0	14.5	《中国药典》2020年版四部通则2201	符合
【含量测定】					
松脂醇二葡萄糖苷	%	≥0.10	0.13	《中国药典》2020年版四部通则0512	符合
检验结论	本品按《中国药典》2020年版一部及四部检验，结果符合规定。				
备　　注	带“*”的检测项目为引用中药材检验数据；报告以加盖检测中心检验报告专用章的纸质报告书为准				

---报告结束---

检验人：　　　复核人：　　　签发人：

Epimedium brevicornu

康美药业股份有限公司检测中心
检验报告

报告书编号：BM202305240005　　　　　　　　KM-CX02205-01

检品名称	淫羊藿	产　　地	甘肃
检品来源	车间	包装规格	统装
数　　量	/	批　　号	230501431
取 样 量	210g	请 检 人	许木书
检验目的	入库检验	生产日期	/
请检日期	2023 年 05 月 20 日	报告日期	2023 年 05 月 24 日
检验依据	《中国药典》2020 年版一部		

检验项目	标准规定	检验结果	结论
【性状】	应具有淫羊藿的性状特征	具有淫羊藿的性状特征	符合规定
【鉴别】			
薄层鉴别*	供试品色谱中，在与对照品色谱相应的位置上，应显相同的暗红色斑点；喷以三氯化铝试液，再置紫外光灯（365nm）下检视，应显相同的橙红色荧光斑点	供试品色谱中，在与对照品色谱相应的位置上，显相同的暗红色斑点；喷以三氯化铝试液，再置紫外光灯（365nm）下检视，显相同的橙红色荧光斑点	符合规定
【检查】			
药屑、杂质	不得过 3%	0.3%	符合规定
水分	不得过 12.0%	9.3%	符合规定
总灰分*	不得过 8.0%	7.6%	符合规定
二氧化硫残留量*	不得过 150mg/kg	未检出	符合规定
【浸出物】*	不得少于 15.0%	30.8%	符合规定
【含量测定】			
总黄酮*	本品按干燥品计算，含总黄酮以淫羊藿苷（$C_{33}H_{40}O_{15}$）计，不得少于 5.0%	8.2%	符合规定
总黄酮醇苷*	本品按干燥品计算，叶片含朝藿定 A（$C_{39}H_{50}O_{20}$）、朝藿定 B（$C_{38}H_{48}O_{19}$）．朝藿定 C（$C_{39}H_{50}O_{19}$）和淫羊藿苷（$C_{33}H_{40}O_{15}$）的总量，朝鲜淫	1.6%	符合规定
检验结论	本品按《中国药典》2020 年版一部检验，结果符合规定。		
备　　注	带"*"的检测项目为引用中药材检验数据；报告以加盖检测中心检验报告专用章的纸质报告书为准		

检验人：　　　　复核人：　　　　签发人：

Epimedium brevicornu

文件编码：KM-CX02205-02

康美药业股份有限公司检测中心
检验报告

报告编号：BM202506140003　　　　共1页，第1页

产品名称	淫羊藿	药材产地	甘肃陇南
装　　量	3g/5g/6g/10g/12g/15g/20g/25g/30g/50g/60g/100g/250g/500g/600g/1kg/2kg/3kg/统装等		
规　　格	丝	产品批号	250505051
取 样 量	200g	样品状态	完好
检验目的	入库检验	样品编号	20250609025
收样日期	2025年06月09日	报告/签发日期	2025年06月14日
实验活动日期	2025年06月09日至2025年06月14日		
检验依据	《中国药典》2020年版一部及四部		

检测项目	单位	评定指标	检测结果	检测方法	单项评定
【性状】	/	具有淫羊藿的性状特征	符合规定	《中国药典》2020年版四部通则0212	符合
【鉴别】					
薄层鉴别*	/	置紫外光灯（365nm）下检视，供试品色谱中，在与对照品色谱相应的位置上，显相同的暗红色斑点；喷以三氯化铝试液，再置紫外光灯（365nm）下检视，显相同的橙红色荧光斑点	符合规定	《中国药典》2020年版四部通则0502	符合
【检查】					
药屑、杂质	%	≤3	0.9	《中国药典》2020年版四部通则2301	符合
水分	%	≤12.0	9.0	《中国药典》2020年版四部通则0832	符合
总灰分*	%	≤8.0	7.0	《中国药典》2020年版四部通则2302	符合
二氧化硫残留量*	mg/kg	≤150	未检出（检出限10）	《中国药典》2020年版四部通则2331	符合
【浸出物】*	%	≥15.0	33.1	《中国药典》2020年版四部通则2201	符合
【含量测定】					
总黄酮*	%	≥5.0	9.5	《中国药典》2020年版四部通则0401	符合
总黄酮醇苷*	%	≥0.50	1.65	《中国药典》2020年版四部通则0512	符合
检验结论	本品按《中国药典》2020年版一部及四部检验，结果符合规定。				
备　　注	带"*"的检测项目为引用中药材检验数据；报告以加盖检测中心检验报告专用章的纸质报告书为准				

---报告结束---

检验人：　　　　复核人：　　　　签发人：

Epimedium brevicornu

文件编码：KM-CX02205-02

康美药业股份有限公司检测中心
检验报告

报告编号：BM202412310003　　　　共1页，第1页

产品名称	淫羊藿	药材产地	甘肃陇南
装　　量	3g/5g/6g/10g/12g/15g/20g/25g/30g/50g/60g/100g/250g/500g/600g/1kg/2kg/3kg/统装等		
规　　格	丝	产品批号	241203751
取 样 量	200g	样品状态	完好
检验目的	入库检验	样品编号	20241227049
收样日期	2024年12月27日	报告/签发日期	2024年12月31日
实验活动日期	2024年12月27日至2024年12月31日		
检验依据	《中国药典》2020年版一部及四部		

检测项目	单位	评定指标	检测结果	检测方法	单项评定
【性状】	/	具有淫羊藿的性状特征	符合规定	《中国药典》2020年版四部通则0212	符合
【鉴别】					
薄层鉴别*	/	置紫外光灯（365nm）下检视，供试品色谱中，在与对照品色谱相应的位置上，显相同的暗红色斑点；喷以三氯化铝试液，再置紫外光灯（365nm）下检视，显相同的橙红色荧光斑点	符合规定	《中国药典》2020年版四部通则0502	符合
【检查】					
药屑、杂质	%	≤3	0.3	《中国药典》2020年版四部通则2301	符合
水分	%	≤12.0	6.8	《中国药典》2020年版四部通则0832	符合
总灰分*	%	≤8.0	5.6	《中国药典》2020年版四部通则2302	符合
二氧化硫残留量*	mg/kg	≤150	未检出（检出限10）	《中国药典》2020年版四部通则2331	符合
【浸出物】*	%	≥15.0	28.1	《中国药典》2020年版四部通则2201	符合
【含量测定】					
总黄酮*	%	≥5.0	7.6	《中国药典》2020年版四部通则0401	符合
总黄酮醇苷*	%	≥0.50	2.8	《中国药典》2020年版四部通则0512	符合
检验结论	本品按《中国药典》2020年版一部及四部检验，结果符合规定。				
备　　注	带“*”的检测项目为引用中药材检验数据；报告以加盖检测中心检验报告专用章的纸质报告书为准				

---报告结束---

检验人：[illegible]　　复核人：[illegible]　　签发人：[illegible]

北京康美制药有限公司

药品检验报告

编号：REC-QP021－02

报告书编号：2023071301

检品名称	白术	产　地	浙江
检品来源	待包装产品库	批　号	230560851
包装规格	统装	取样量	200g
数　量	15076.3kg	请检人	量传霞
检验目的	质量检验	请检日期	2023年07月08日
有效期	无	报告日期	2023年07月13日
检验依据	《中国药典》2020年版一部及四部		

检验项目	标准规定	检验结果	结论
【性状】	应与白术相符	与白术相符	符合规定
【鉴别】			
薄层鉴别	供试品主斑点的位置与颜色应与白术对照药材相同	相同	符合规定
【检查】			
药屑杂质	不得过3%	0.5%	符合规定
水分	不得过15.0%	12.9%	符合规定
总灰分	不得过5.0%	3.9%	符合规定
色度	不得更深	未更深	符合规定
二氧化硫残留量	不得过400mg/kg	0mg/kg	符合规定
【浸出物】	不得少于35.0%	43.5%	符合规定

结论：本品按《中国药典》2020年版一部及四部检验，结果符合规定。

检验人：[illegible]　　复核人：[illegible]　　质量控制负责人：[illegible]

Atractylodes macrocephala

岭南中药饮片有限公司

成品检验报告书

报告书编号：Cg-015-01-07-2504001　　　　SOR-ZJ-009-01

检品名称	白术	产地	浙江金华
批号	2504001	规格	片；选货
数量	5044.4kg	检品来源	包装车间
物料编码	Cg-015-01-07	生产日期	2025年04月10日
取样量	300g	请检日期	2025年05月12日
检验目的	入库检验	报告日期	2025年05月14日
装量	3g, 6g, 5g, 10g, 15g, 100g, 250g, 0.25kg, 0.5kg, 1kg, 2kg, 25kg, 0.1kg, 选装		
检验依据	《中国药典》2020年版一部"白术"质量标准		

检验项目	标准规定	检验结果	结论
【性状】	应具标准规定的性状特征	具标准规定的性状特征	符合规定
【鉴别】薄层鉴别	供试品色谱中，在与对照药材色谱相应的位置上，应显相同颜色的斑点，并应显有一桃红色主斑点(苍术酮)。	供试品色谱中，在与对照药材色谱相应的位置上，显相同颜色的斑点，并显有一桃红色主斑点(苍术酮)。	符合规定
【检查】			
杂质	不得过3%	0.1%	符合规定
水分	不得过15.0%	12.1%	符合规定
总灰分	不得过5.0%	3.7%	符合规定
色度	应符合规定	符合规定	符合规定
二氧化硫残留量	不得过400mg/kg	＜10mg/kg	符合规定
33种禁用农药	不得检出	符合规定	符合规定
【浸出物】	不得少于35.0%。	41.4%	符合规定

以下空白

质量管理专用章 (3)

岭南中药饮片有限公司 质量部

岭南中药饮片有限公司 质检部

检验结论	本品按《中国药典》2020年版一部"白术"质量标准检验，结果 符合 规定。
备注	检验结果引用白术 2504001 待包装产品检验数据。

检验人：[illegible]　　复核人：[illegible]　　负责人：[illegible]

Atractylodes macrocephala

康美（亳州）世纪国药有限公司

检验报告单

REC-ZL-KB-059-01

报告单编号：C010-2406003　　　**报告日期：2024 年 06 月 24 日**

品　　名	白术	数　　量	13956kg
批号/编号	240600109	产　　地	安徽 亳州
规　　格	统	检品来源	饮片车间
请验日期	2024 年 06 月 24 日	检验目的	成品入库
检验依据	《中国药典》2020 年版一部、四部		

检验项目	标准规定	检验结果
【性状】	应具有白术的性状特征	具有白术的性状特征
【鉴别】		
薄层鉴别	供试品色谱中，在与白术对照药材色谱相应的位置上，应显相同颜色的斑点，并应显有一桃红色主斑点（苍术酮）	与白术对照药材色谱一致
【检查】		
药屑杂质	应不得过 3%	0.6%
水分	应不得过 15.0%	10.6%
总灰分	应不得过 5.0%	4.1%
二氧化硫残留量	应不得过 400mg/kg	238mg/kg
色度	应符合规定	符合规定
【浸出物】		
醇溶性浸出物	应不得少于 35.0%	66.0%

结论	本品按《中国药典》2020 年版一部、四部检验，结果符合规定。
备注	性状、鉴别、检查、浸出物引取中间产品白术 240600109 批送检结果。

质量负责人：[illegible]　　　复核人：[illegible]　　　检验人：[illegible]

Plantago asiatica

GDYZ-QR-25-04 A.0

检测报告

报告编号：RC230706008

样品名称		车前草	样品批号	23040204
样品编号		C230706005	产品代码	CC061
规　　格		500g/袋	批　　量	346 袋
产　　地		河南	生产日期	2023 年 04 月 21 日
请检日期		2023 年 07 月 06 日	报告日期	2023 年 07 月 06 日
生产单位		广东云智中药饮片有限公司		
检验依据		《中华人民共和国药典》2020 年版、<br>《车前草成品内控质量标准》TS-QA-CC061-00		
检验项目		质量标准		检验结果
性状		应符合标准规定		符合规定
鉴别	显微鉴别	应符合标准规定		符合规定
	薄层鉴别	应符合标准规定		符合规定
检查	药屑及杂质	≤3.0%		0.7%
	水分	≤13.0%		11.4%
	总灰分	≤15.0%		14.4%
	酸不溶性灰分	≤5.0%		3.2%
	二氧化硫残留量	≤150mg/kg		未检出
浸出物		不得少于 14.0%		40.5%
含量测定		本品按干燥品计算，含大车前苷（[illegible]）<br>不得少于 0.10%		0.82%
（以下空白）				
检验结论		本品按照《中华人民共和国药典》2020 年版、《车前草成品内控质量标准》YS-QA-CC061-00 检验，检验结果符合规定。		
备注		本批次检验结果数据引用为车前草成品 20230402 [illegible]成品检验报告书编号为：RC230519005。		

编制人：[illegible]　　复核人：[illegible]　　批准人：[illegible]

Plantago asiatica

中山市仙逸堂中药饮片有限公司

成品检验报告

报告编号：BSC2411243 文件编号：REC-QF02027-02

品　名	车前草	批　号	2411243
装　量	1000g/袋	检品数量	200g
产　地	江西吉安	产品来源	生产车间
生产日期	2024年11月28日	检验项目	全检
检验目的	成品检验	收检日期	2024年12月03日
检验依据	《中国药典》2020年版一部、四部	报告日期	2024年12月04日

检验项目	标准规定	检验结果
【性状】	本品为不规则的段。根须状或直而长。叶片皱缩，多破碎，表面灰绿色或污绿色，脉明显。可见穗状花序，气微，味微苦。	符合规定
【鉴别】		
1. 显微鉴别	应符合规定	符合规定
2. 薄层鉴别	供试品色谱中，在与对照品色谱相应的位置上，显相同颜色的斑点。	符合规定
【检查】		
1. 药屑、杂质	应不得过3.0%	0.2%
2. 水分	应不得过13.0%	10.4%
3. 总灰分	应不得过15.0%	12.8%
4. 酸不溶性灰分	应不得过5.0%	3.8%
5. 二氧化硫残留量	应不得过150mg/kg	0mg/kg
【浸出物】	应不得少于14.0%	30.8%
【含量测定】	本品按干燥品计算，含大车前苷（$C_{29}H_{36}O_{16}$）不得少于0.10%。	0.21%
【33种禁用农药】	应不得检出	未检出

以下空白

备注	以上项目（除性状、水分外）引用车前草待包装产品（2411243）检验结果。				
检验结论	本品按《中国药典》2020年版一部、四部检验，结果符合规定。				
检验员	杨悦	复核员	[illegible]	受权人或转受权人	[illegible]

Plantago asiatica

岭南中药饮片有限公司

成品检验报告书

报告书编号：Cc-034-01-07-2505001　　　　　　SOR-ZJ-009-01

检品名称	车前草	产地	广东清远
批号	2505001	规格	段
数量	1826kg	检品来源	包装车间
物料编码	Cc-034-01-07	生产日期	2025 年 05 月 18 日
取样量	300g	请检日期	2025 年 06 月 05 日
检验目的	入库检验	报告日期	2025 年 06 月 11 日
装量	3g, 6g, 5g, 10g, 15g, 100g, 250g, 0.25kg, 0.5kg, 1kg, 2kg, 25kg, 0.1kg, 选装		
检验依据	《中国药典》2020 年版一部“车前草”质量标准		

检验项目	标准规定	检验结果	结论
【性状】	应具标准规定的性状特征	具标准规定的性状特征	符合规定
【鉴别】<br>显微鉴别	应符合规定	符合规定	符合规定
薄层鉴别	供试品色谱中，在与对照品色谱相应的位置上，应显相同颜色的斑点。	供试品色谱中，在与对照品色谱相应的位置上，显相同颜色的斑点。	符合规定
【检查】<br>杂质	不得过 3%	0.1%	符合规定
水分	不得过 13.0%	12.0%	符合规定
总灰分	不得过 15.0%	12.3%	符合规定
酸不溶性灰分	不得过 5.0%	1.7%	符合规定
二氧化硫残留量	不得过 150mg/kg	＜10mg/kg	符合规定
【浸出物】	不得少于 14.0%	32.9%	符合规定
【含量测定】	本品按干燥品计算，含大车前苷 ($C_{29}H_{36}O_{16}$)不得少于 0.10%。	0.28%	符合规定

以下空白

检验结论	本品按《中国药典》2020 年版一部“车前草”质量标准检验，结果 符合 规定。
备注	检验结果引用车前草 2505001 待包装产品检验数据。

检验人：　　　复核人：　　　负责人：

Clematis chinensis

文件编码：KM-CX02205-02

康美药业股份有限公司检测中心
检验报告

报告编号：BM202506100014　　　　共1页，第1页

产品名称	威灵仙	药材产地	辽宁本溪
装　　量	3g/5g/6g/10g/12g/15g/20g/25g/30g/50g/60g/100g/250g/500g/600g/1kg/2kg/3kg/统装等		
规　　格	段	产品批号	250503681
取 样 量	200g	样品状态	完好
检验目的	入库检验	样品编号	20250604023
收样日期	2025年06月04日	报告/签发日期	2025年06月10日
实验活动日期	2025年06月04日至2025年06月10日		
检验依据	《中国药典》2020年版一部及四部		

检测项目	单位	评定指标	检测结果	检测方法	单项评定
【性状】	/	具有威灵仙的性状特征	符合规定	《中国药典》2020年版四部通则0212	符合
【鉴别】					
薄层鉴别*	/	供试品色谱中，在与对照品色谱相应的位置上，显相同颜色的斑点	符合规定	《中国药典》2020年版四部通则0502	符合
【检查】					
药屑、杂质	%	≤3	0.5	《中国药典》2020年版四部通则2301	符合
水分	%	≤15.0	8.6	《中国药典》2020年版四部通则0832	符合
总灰分*	%	≤10.0	9.3	《中国药典》2020年版四部通则2302	符合
酸不溶性灰分*	%	≤4.0	3.3		符合
二氧化硫残留量*	mg/kg	≤150	未检出（检出限10）	《中国药典》2020年版四部通则2331	符合
【浸出物】	%	≥15.0	23.5	《中国药典》2020年版四部通则2201	符合
【含量测定】					
齐墩果酸	%	≥0.30	1.57	《中国药典》2020年版四部通则0512	符合
检验结论	本品按《中国药典》2020年版一部及四部检验，结果符合规定。				
备　　注	带“*”的检测项目为引用中药材检验数据；报告以加盖检测中心检验报告专用章的纸质报告书为准				

---报告结束---

检验人：　　　　复核人：　　　　签发人：

Clematis chinensis

文件编码：KM-CX02205-02

康美药业股份有限公司检测中心
检验报告

报告编号：BM202407180013 　　　　共1页，第1页

检品名称	威灵仙	产　地	辽宁本溪
规　　格	3g/5g/6g/10g/12g/15g/20g/25g/30g/50g/60g/100g/250g/500g/600g/1kg/2kg/3kg/统装等		
请 检 人	方益隆	批　号	240604601
取 样 量	210g	样品状态	完好
检验目的	入库检验	样品编号	20240712032
收样日期	2024年07月12日	报告/签发日期	2024年07月18日
实验活动日期	2024年07月14日至2024年07月18日		
检验依据	《中国药典》2020年版一部及四部		

检测项目	单位	评定指标	检测结果	检测方法	单项评定
【性状】	/	具有威灵仙的性状特征	符合规定	《中国药典》2020年版四部通则0212	符合
【鉴别】					
薄层鉴别*	/	供试品色谱中，在与对照品色谱相应的位置上，显相同颜色的斑点	符合规定	《中国药典》2020年版四部通则0502	符合
【检查】					
药屑、杂质	%	≤3	0.4	《中国药典》2020年版四部通则2301	符合
水分	%	≤15.0	8.0	《中国药典》2020年版四部通则0832	符合
总灰分*	%	≤10.0	6.21	《中国药典》2020年版四部通则2302	符合
酸不溶性灰分*	%	≤4.0	2.4		符合
二氧化硫残留量*	mg/kg	≤150	未检出（检出限10）	《中国药典》2020年版四部通则2331	符合
【浸出物】	%	≥15.0	21.1	《中国药典》2020年版四部通则2201	符合
【含量测定】					
齐墩果酸	%	≥0.30	1.4	《中国药典》2020年版四部通则0512	符合
检验结论	本品按《中国药典》2020年版一部及四部检验，结果符合规定。				
备　　注	带"*"的检测项目为引用中药材检验数据；报告以加盖检测中心检验报告专用章的纸质报告书为准				

---报告结束---

检验人：[illegible]　　复核人：[illegible]　　签发人：[illegible]

Clematis chinensis

康美药业股份有限公司检测中心
检验报告

报告书编号：BM202306200029　　　　　　　　KM-CX02205-01

检品名称	威灵仙	产　　地	辽宁
检品来源	车间	包装规格	统装
数　　量	/	批　　号	230505401
取 样 量	210g	请 检 人	李楚边
检验目的	入库检验	生产日期	/
请检日期	2023 年 06 月 13 日	报告日期	2023 年 06 月 20 日
检验依据	《中国药典》2020 年版一部		

检验项目	标准规定	检验结果	结论
【性状】	应具有威灵仙的性状特征	具有威灵仙的性状特征	符合规定
【鉴别】			
薄层鉴别*	供试品色谱中，在与对照品色谱相应的位置上，应显相同颜色的斑点	供试品色谱中，在与对照品色谱相应的位置上，显相同颜色的斑点	符合规定
【检查】			
药屑、杂质	不得过 3%	0.5%	符合规定
水分	不得过 15.0%	6.1%	符合规定
总灰分*	不得过 10.0%	7.13%	符合规定
酸不溶性灰分*	不得过 4.0%	3.0%	符合规定
二氧化硫残留量*	不得过 150mg/kg	未检出	符合规定
【浸出物】	不得少于 15.0%	16.6%	符合规定
【含量测定】	本品按干燥品计算，含齐墩果酸（$C_{30}H_{48}O_3$）不得少于 0.30%	1.3%	符合规定

注：二氧化硫残留量检出限：二氧化硫残留量小于 10mg/kg 视为未检出或 0mg/kg。

以下空白

检验结论	本品按《中国药典》2020 年版一部检验，结果符合规定。
备　　注	带"*"的检测项目为引用中药材检验数据；报告以加盖检测中心检验报告专用章的纸质报告书为准

检验人：[illegible]　　复核人：[illegible]　　签发人：[illegible]

Morinda officinalis

文件编码：KM-CX02205-02

康美药业股份有限公司检测中心
检验报告

报告编号：BM202506230020　　　　共1页，第1页

产品名称	盐巴戟天	药材产地	广东肇庆
装　　量	3g/5g/6g/10g/12g/15g/20g/25g/30g/50g/60g/100g/250g/500g/600g/1kg/2kg/3kg/统装等		
规　　格	段	产品批号	250504911
取 样 量	200g	样品状态	完好
检验目的	入库检验	样品编号	20250615010
收样日期	2025年06月15日	报告/签发日期	2025年06月23日
实验活动日期	2025年06月15日至2025年06月23日		
检验依据	《中国药典》2020年版一部及四部		

检测项目	单位	评定指标	检测结果	检测方法	单项评定
【性状】	/	具有盐巴戟天的性状特征	符合规定	《中国药典》2020年版四部通则0212	符合
【鉴别】					
薄层鉴别*	/	供试品色谱中，在与对照药材色谱相应的位置上，显相同颜色的斑点	符合规定	《中国药典》2020年版四部通则0502	符合
【检查】					
药屑、杂质	%	≤3	0.6	《中国药典》2020年版四部通则2301	符合
水分	%	≤15.0	9.2	《中国药典》2020年版四部通则0832	符合
总灰分	%	≤8.0	3.4	《中国药典》2020年版四部通则2302	符合
二氧化硫残留量*	mg/kg	≤150	未检出（检出限10）	《中国药典》2020年版四部通则2331	符合
【浸出物】	%	≥50.0	81.5	《中国药典》2020年版四部通则2201	符合
【含量测定】					
耐斯糖	%	≥2.0	7.1	《中国药典》2020年版四部通则0512	符合
检验结论	本品按《中国药典》2020年版一部及四部检验，结果符合规定。				
备　　注	带"*"的检测项目为引用中药材检验数据；报告以加盖检测中心检验报告专用章的纸质报告书为准				

---报告结束---

检验人：[illegible]　　　　复核人：[illegible]　　　　签发人：[illegible]

Morinda officinalis

康美药业股份有限公司检测中心
检验报告

报告书编号：BM202307100013　　　　KM-CX02205-01

检品名称	盐巴戟天	产　　地	广东
检品来源	车间	包装规格	统装
数　　量	/	批　　号	230603011
取 样 量	210g	请 检 人	郑庐发
检验目的	入库检验	生产日期	/
请检日期	2023 年 07 月 03 日	报告日期	2023 年 07 月 10 日
检验依据	《中国药典》2020 年版一部		
检验项目	标准规定	检验结果	结论
【性状】	应具有盐巴戟天的性状特征	具有盐巴戟天的性状特征	符合规定
【鉴别】<br>薄层鉴别*	供试品色谱中，在与对照药材色谱相应的位置上，应显相同颜色的斑点	供试品色谱中，在与对照药材色谱相应的位置上，显相同颜色的斑点	符合规定
【检查】			
药屑、杂质	不得过 3%	0.3%	符合规定
水分	不得过 15.0%	6.8%	符合规定
总灰分	不得过 8.0%	5.5%	符合规定
二氧化硫残留量*	不得过 150mg/kg	未检出	符合规定
【浸出物】	不得少于 50.0%	80.6%	符合规定
【含量测定】	本品按干燥品计算，含耐斯糖（$C_{24}H_{42}O_{21}$）不得少于 2.0%	5.1%	符合规定

注：二氧化硫残留量检出限：二氧化硫残留量小于 10mg/kg 视为未检出或 0mg/kg。

以下空白

检验结论	本品按《中国药典》2020 年版一部检验，结果符合规定。
备　　注	带“*”的检测项目为引用中药材检验数据；报告以加盖检测中心检验报告专用章的纸质报告书为准

检验人：[illegible]　　复核人：[illegible]　　签发人：[illegible]

Morinda officinalis

文件编码：KM-CX02205-02

康美药业股份有限公司检测中心
检验报告

报告编号：BM202407120030　　　　共1页，第1页

检品名称		盐巴戟天	[illegible]地	广东肇庆	
规　　格		3g/5g/6g/10g/12g/15g/20g/25g/30g/50g/60g/100g/250g/500g/600g/1kg/2kg/3kg/统装等			
请 检 人		王正兵	批　　号	240603871	
取 样 量		210g	样品状态	完好	
检验目的		入库检验	样品编号	20240704019	
收样日期		2024年07月04日	报告/签发日期	2024年07月12日	
实验活动日期		2024年07月05日至2024年07月12日			
检验依据		《中国药典》2020年版一部及四部			
检测项目	单位	评定指标	检测结果	检测方法	单项评定
【性状】	/	具有盐巴戟天的性状特征	符合规定	《中国药典》2020年版四部通则0212	符合
【鉴别】					
薄层鉴别*	/	供试品色谱中，在与对照药材色谱相应的位置上，显相同颜色的斑点	符合规定	《中国药典》2020年版四部通则0502	符合
【检查】					
药屑、杂质	%	≤3	0.1	《中国药典》2020年版四部通则2301	符合
水分	%	≤15.0	11.1	《中国药典》2020年版四部通则0832	符合
总灰分	%	≤8.0	4.0	《中国药典》2020年版四部通则2302	符合
二氧化硫残留量*	mg/kg	≤150	未检出（检出限10）	《中国药典》2020年版四部通则2331	符合
【浸出物】	%	≥50.0	77.2	《中国药典》2020年版四部通则2201	符合
【含量测定】					
耐斯糖	%	≥2.0	6.1	《中国药典》2020年版四部通则0512	符合
检验结论	本品按《中国药典》2020年版一部及四部检验，结果符合规定。				
备　　注	带“*”的检测项目为引用中药材检验数据；报告以加盖检测中心检验报告专用章的纸质报告书为准				

——报告结束——

检验人：[illegible]　　　　复核人：[illegible]　　　　签发人：[illegible]

Figure S1. Botanical drug quality report

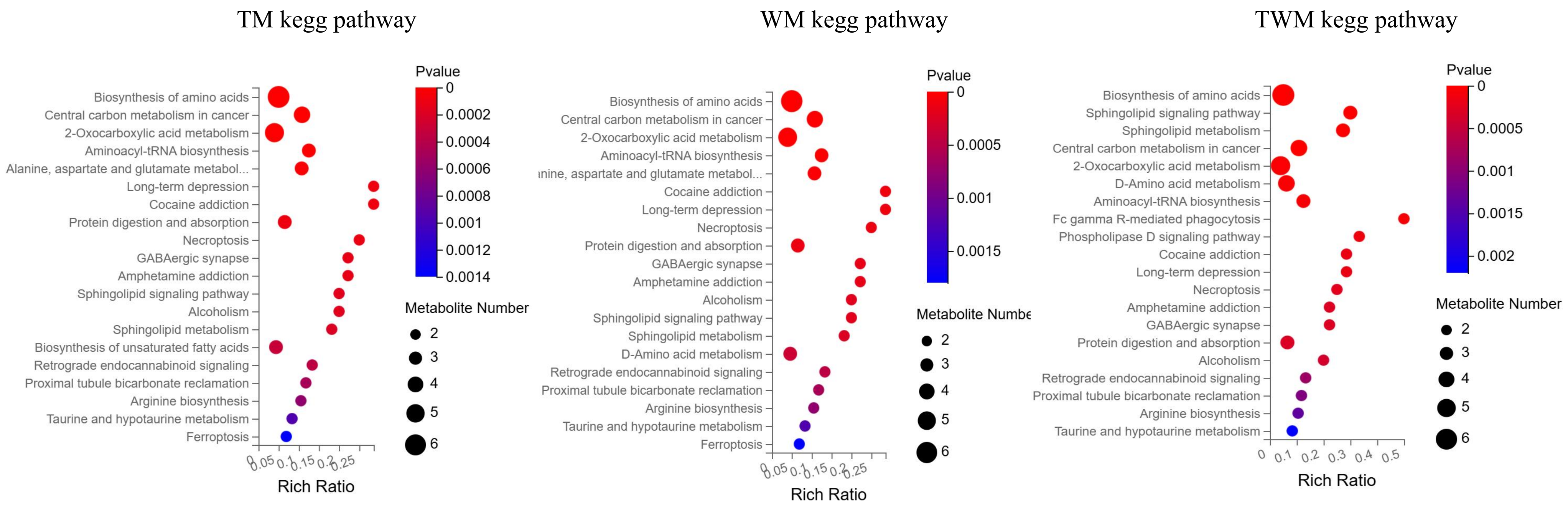

Figure S2. KEGG pathway enrichment of DEMs in three groups.

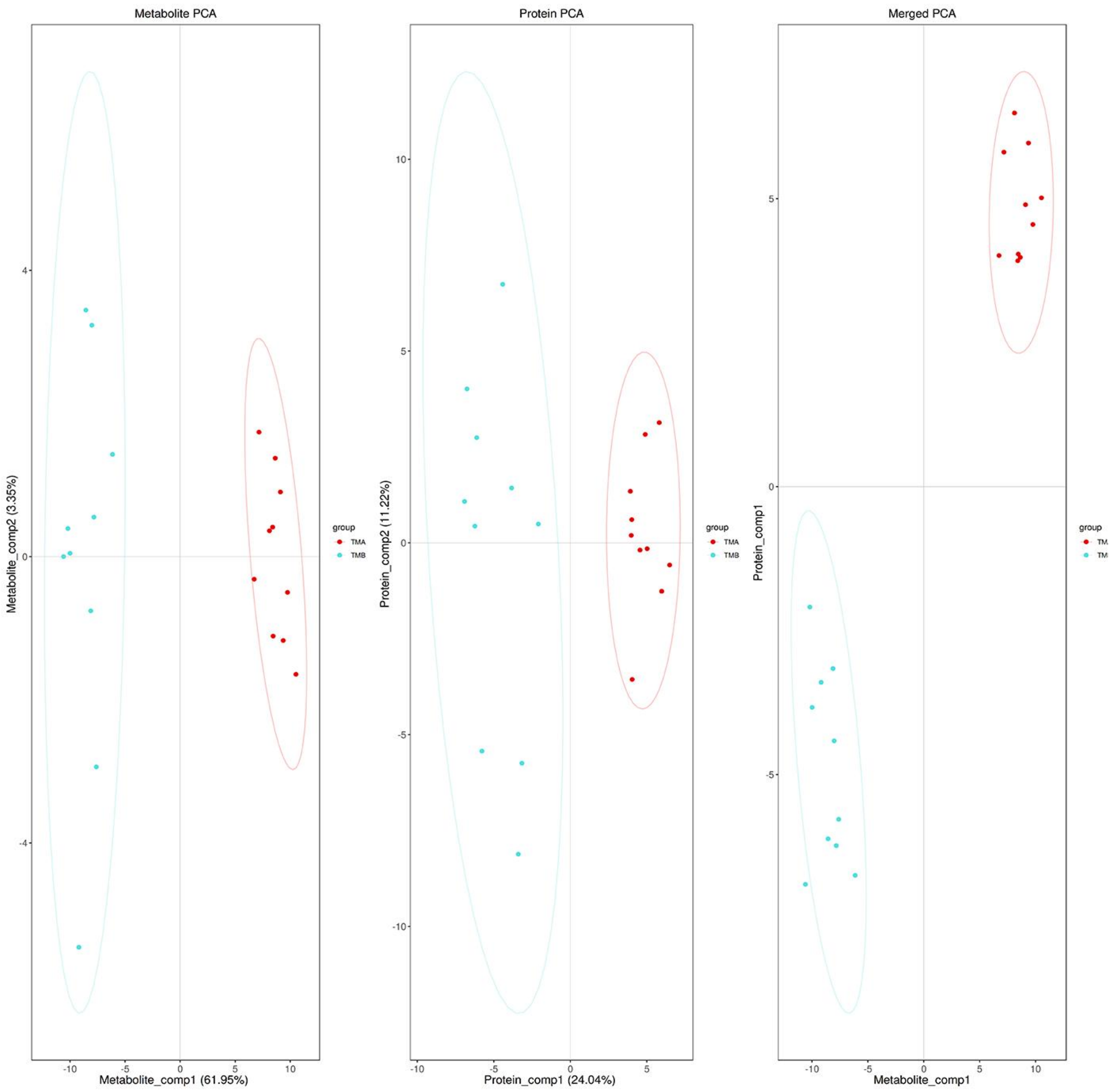

Figure S3. PCA score plots of pre- and post-treatment samples among the three groups (TM).

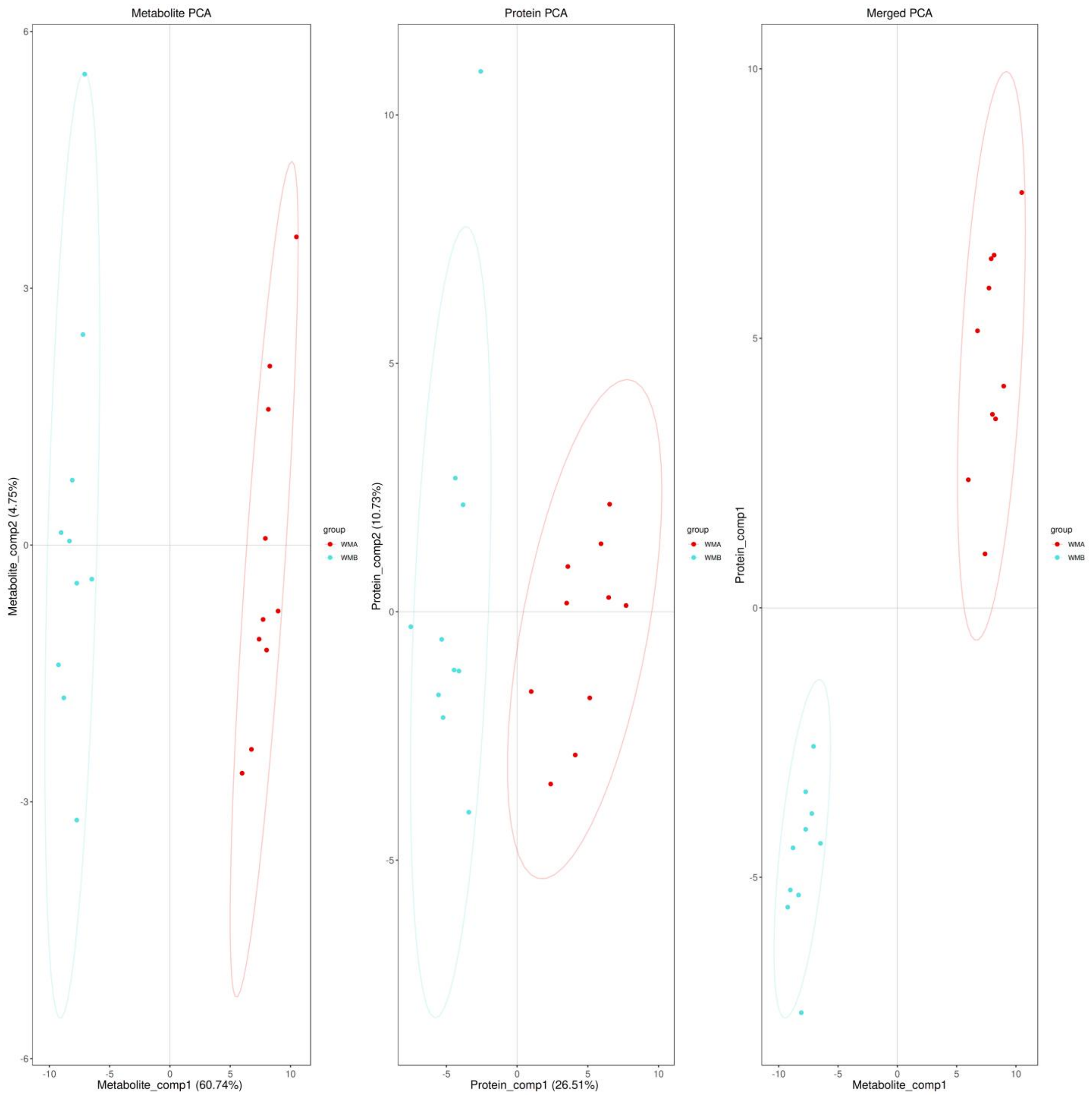

Figure S3. (Continued.) PCA score plots of pre- and post-treatment samples among the three groups (WM).

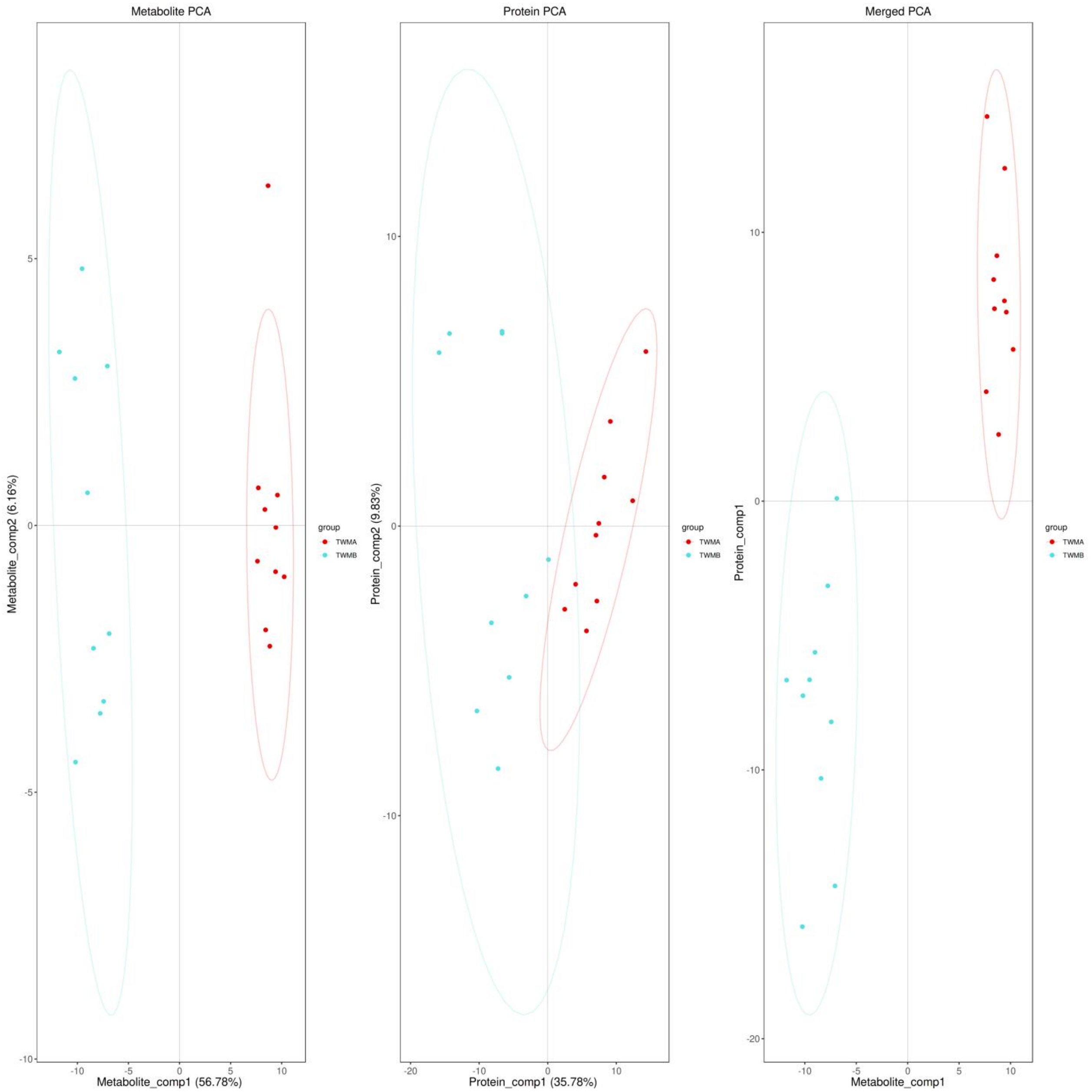

Figure S3. (Continued.) PCA score plots of pre- and post-treatment samples among the three groups (TWM).
